# The surge of earthquakes in Central Oklahoma has features of reservoir-induced seismicity

**DOI:** 10.1038/s41598-018-29883-9

**Published:** 2018-07-31

**Authors:** Lisa Johann, Serge A. Shapiro, Carsten Dinske

**Affiliations:** Freie Universitaet Berlin, Institute of Geophysics, Berlin, 12249 Germany

## Abstract

The recent surge of seismicity in Oklahoma and Kansas is related to fluid disposal. Evidences suggest that critical parameters are the injection volume as well as injection depth but dominant physical processes and a corresponding model to describe the physics are still not clear. We analyse the spatio-temporal distribution of induced earthquakes in the basement and find visible signatures of pore pressure diffusion and poroelastic coupling, features which strongly resemble seismicity induced by the filling of artificial lakes, so-called reservoir-induced seismicity. We developed a first-principle model of underground reservoir-induced seismicity. The physics of the model are based upon the combined mechanisms of fluid mass added to the pore-space of the injection layer and acting as a normal stress on the basement surface, pore-fluid pressure diffusion in the basement as well as poroelastic coupling contributing to the pore-fluid pressure and stress. Furthermore, we demonstrate that underground reservoir-induced seismicity occurs preferably in normal faulting and strike-slip settings, the latter being prevalent in Oklahoma. Our model explains observed injection volume and depth dependence of the seismicity and should be considered as a basis for future hazard prediction and prevention as well as for planning possible disposal sites.

## Introduction

Starting in 2009, an unexpected burst of earthquakes has struck the central U.S.^1,2^. Whereas only about one magnitude *M* ≥ 3 earthquake happened per year in north-central Oklahoma before 2009, approximately 900 *M* ≥ 3 events were recorded in 2015^3^. It is now widely understood that this acceleration of seismic activity is linked to the injection of huge volumes of waste water through salt water disposal (SWD) wells^2,3^. Most of these wells inject into the highly permeable, underpressured Arbuckle aquifer which is hydraulically connected to the underlying crystalline basement where most of the seismicity occurs. In reaction to the strong increase of earthquakes, the Oklahoma Corporation Commission (OCC) Oil and Gas Division called for a 40% reduction of the 2014 injection volume in Central Oklahoma to be completed in mid- 2016.

Numerous studies on mechanisms explaining the spatio-temporal evolution of the observed fluid-disposal induced seismicity have been published to date. There are indications that the injection volume as well as injection depth affect the seismic activity^4^. However, it remains a challenging task to assess the governing physical processes because they are assumed to deviate from the ones which control seismicity induced by high-pressure reservoir stimulations^5,6^. For the case of Oklahoma, firstly, events occur in the deeper basement and not directly in the overlying injection formation. Secondly, seismicity is also observed over broad areas far from injectors. And thirdly, unlike in the case of pure pore-fluid pressure diffusion where the spatio-temporal event evolution is enveloped by a triggering front^7^, the time and location of earthquakes in Oklahoma does not clearly obey such a behaviour^8^. Published models include pore-fluid pressure diffusion^2,3^ as well as poroelastic fluid-solid coupling effects^8–11^. Yet, the controlling mechanisms of seismic activity in Oklahoma are still not fully understood. Since the number of damaging earthquakes poses a risk not only to infrastructure and buildings but also to human life, a model capable of explaining spatio-temporal features of the seismicity is fundamental for seismic hazard mitigation.

Considering the scenario of large-volume waste water disposal and using knowledge of the concept of seismicity induced by the filling of surface water reservoirs, known as reservoir-induced seismicity (RIS)^12–14^, we developed a new first-principle model called *underground reservoir-induced seismicity* (URIS). Our studies demonstrate that such a model is able to capture the spatio-temporal evolution of the observed seismicity in Central Oklahoma. To draw the connection between URIS and RIS, we assume that the rapid increase of fluid disposal rates in the highly permeable Arbuckle formation corresponds to the filling of a large subsurface reservoir. As a consequence, pressure and stress changes in the underlying basement are observed. Accepting that the basement acts as a poroelastic half-space, it is a combination of physical mechanisms that result in pressure- and stress changes in the basement. These mechanisms are the direct effect of mass added to the injection formation (here the Arbuckle aquifer), pore-fluid pressure diffusion in the crystalline basement as well as poroelastic coupling.

We derive an analytical solution for the corresponding initial value poroelastic uniaxial strain problem with constant boundary conditions. Our derivation is directly based on previously elaborated approaches for poroelastic effects of injections and RIS effects^14–26^. Using hydrological and elastic parameters for Oklahoma from literature, analytical pore-fluid pressure and stress solutions are compared to results obtained by finite element modelling. As the ambient stress and pressure states influence the occurrence of RIS^12^, we transfer this knowledge to the case of URIS by computing the change in failure criterion stress for different tectonic stress regimes. We then account for the time of fluid accumulation in the Arbuckle formation by defining a time-dependent boundary condition for the pressure and stress acting on the basement top. We solve analytically for such a boundary condition problem, generate synthetic event catalogues for a strike-slip regime and analyse spatio-temporal features of the events. Concluding the work presented here, we compare patterns of synthetic seismicity to Central Oklahoma events.

Our results suggest that, equivalently to RIS, the background stress regime has to be taken into consideration in the URIS model. This is a direct consequence of the poroelastic coupling effect. However, unlike in the case of RIS, filling of an underground reservoir in a thrust faulting regime also induces a destabilisation front which evolves with time from the reservoir bottom. In contrast, in a strike-slip regime, as existing in Central Oklahoma^27^, the domain of destabilisation grows rapidly from the bottom of the Arbuckle formation. A sensitivity study confirmed that the choice of parameters affects the medium destabilisation, yet in the range of general event location uncertainties.

As the application of the URIS model to Oklahoma induced events captures well the depth and time evolution of the observed seismicity, our approach should be considered in future research to reduce the risk posed by the anthropogenic events.

## Results

### Central Oklahoma Seismicity

Recently, Langenbruch and Zoback^3^ found that seismicity in the area of Central Oklahoma started in 2009 after a monthly injection volume threshold of 3.6 × 10^6^ m^3^ had been exceeded. According to the authors, this injection rate sets an upper limit to fluid volumes which can be incorporated into the hydraulic system. If injection rates are higher, *in-situ* stresses are locally modified which in turn might lead to the occurrence of seismic events. The volume reduction plan decreed by the OCC in consequence of the accelerated seismicity rate^3^ appears to have lowered seismicity rates in the period 2015 to the present^28^. Still, there is an ongoing debate on whether or not the probability of larger-magnitude events is also declining^3,8^. Moreover, it was shown that the total seismic moment in Oklahoma has decreased only moderately^4^.

We focus on a catalogue of relocated events published by Schoenball and Ellsworth^29^. This database includes Oklahoma events that occurred between May 2013 and November 2016. Following the hypocentre locations, most of the seismic activity is distributed along previously unknown basement faults and might occur at large distances of up to 40 km from the wells^8,29,30^. This observation points to complex poroelastic coupling effects rather than pure pore-fluid pressure diffusion^8^.

**Figure 1:**
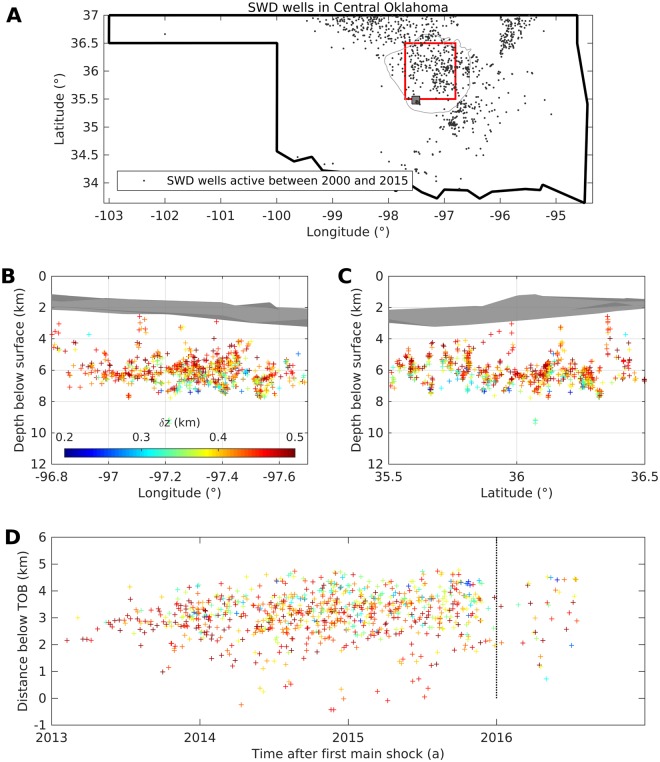
Seismicity in Central Oklahoma from May 2013 to November 2016. The map (**A**) depicts our study area (Central Oklahoma), bounded by latitude 35.5° to 36.5° and longitude −97.7° to −96.8° (red square). Panels (B,C) show event depths (colour-coded by depth uncertainty) and the top of the basement (TOB, grey surface) relatively to the ground surface elevation. In panel (D), event distances below the TOB are plotted versus their occurrence time. Year 0 denotes the time of the first event included in the catalogue on 5 May 2013. Event locations were published by Schoenball and Ellsworth^29^, including only events with vertical errors $\delta z\, < \,$0.5 km and with a magnitude larger than the magnitude of completeness obtained from the Gutenberg-Richter relation (see Supplementary Materials). The depth of the TOB was derived from well data^31^.

We restrict the event catalogue to an area which we define as Central Oklahoma (COH), bounded by longitude [−97.7°, −96.7°] and latitude [35.5°, 36.5°], shown in Fig. 1. Our analysis requires high-precision depth locations, thus we neglect events with depth errors $\delta z\, > \,0.5$ km (Fig. S1). In a later work, Schoenball and Ellsworth^30^ demonstrated that most of the seismicity occurs in sequences with significant fore- and aftershock activity, probably caused by earthquake interaction such as static stress transfer. To exclude these events, we declustered the catalogue (see Supplementary Materials, Fig. S2). As expected, the main shocks now follow a homogeneous Poissonian distribution in time with a magnitude of completeness *M*_*c*_ = 2.4 (Fig. S3). Most of the seismicity with magnitude $M\ge {M}_{c}$ occurs at depths between 5 and 7 km below the surface (Fig. 1). Correlating these depths with the top of the basement (TOB) derived from well data^31^ (grey surface in Fig. 1B,C), hypocentres lie within the upper 2 to 4 km of the basement. With time, the seismogenic zone shifts to greater depths (Figs 1D and S10A).

### Features of Reservoir-Induced Seismicity

More than 70 years ago, Carder^32^ noted a positive correlation between the water level of Lake Mead, Nevada/Arizona (U.S.), and seismicity nearby. In the 1960s, a number of damaging earthquakes occurred in India (Koyna, 1967, *M*6.3), China (Hsingfengkiang, 1962, *M*6.1), Zimbabwe (Kariba, 1963, *M*5.8) and Greece (Kremasta, 1966, *M*6.3), all of which are nowadays understood to be linked to lake level changes^12^. It is widely accepted that the seismicity is induced by perturbations of the ambient stress field. Based on the theory of poroelasticity^15,19,33^, consider a poroelastic half-space extended in the vertical *z*-direction. On the upper boundary, vertical stress and pore-fluid pressure are applied. The stress corresponds to the weight of the water column in the reservoir whereas the pore-fluid pressure is defined by the pressure below the reservoir. Assuming a permeable reservoir bottom, the pressure can diffuse into the pore space of the underlying formation. Clearly, all quantities are functions of the depth *z* and the problem becomes one-dimensional^14,17,18,20,26^. Due to the loading, an instantaneous response of the poroelastic, undrained rocks can be observed in terms of pressure and stress changes. Additionally, pore-fluid pressure diffusion leads to a delayed pressure increase in the basement. Both effects may cause shear failure along pre-existing, favourably oriented and critically stressed fractures^22^.

We here accept the continuum mechanic notation that compressive stresses are negative. Thus, the quantities *σ*_1_, *σ*_2_ and *σ*_3_ are equal to the principal stresses multiplied by (−1) and *σ*_1_ > *σ*_2_ > *σ*_3_, where *σ*_1_, *σ*_2_ and *σ*_3_ denote absolute magnitudes of the maximum, intermediate and minimum principal compressive stress. Following the concept of the failure criterion stress *FCS*:

1 $$FCS=\frac{{\sigma }_{d}}{2}-\,\sin \,\phi ({\sigma }_{m}-p)\,,$$

optimally oriented fractures are activated in the case of *FCS* > 0. Further, destabilisation occurs if the variation Δ*FCS* is positive: ${\rm{\Delta }}FCS=\frac{{\rm{\Delta }}{\sigma }_{d}}{2}-{\rm{s}}{\rm{i}}{\rm{n}}\phi ({\rm{\Delta }}{\sigma }_{m}-{\rm{\Delta }}p) >$ 0 (we assume other parameters, including the cohesion, unaffected). In the above equations, *σ*_*d*_ = *σ*_1_−*σ*_3_ and *σ*_*m*_ = (*σ*_1_ + *σ*_3_)/2 are the differential and mean stress, respectively, and *p* is the pore-fluid pressure. Further, *φ* denotes the angle of internal friction which is related to the friction coefficient *μ*_*f*_ by *μ*_*f*_ = *tanφ*.

Previous works noted that RIS might also occur at large distances from the water in the reservoir. It follows that stress changes in the order of 0.1 MPa are sufficiently high to induce seismic events^34^. Additionally, it was shown that the stress regime near the reservoir significantly affects the occurrence of RIS^12,24^. Theoretically, the increasing vertical stress caused by the filling of the reservoir in a normal faulting regime contributes to the vertically oriented maximum principal stress. This leads to a higher differential stress. In a thrust faulting regime where the vertical stress corresponds to the minimum principal compressive stress, the additional load stabilises locations below the reservoir. This theory has been reviewed for several reservoir locations worldwide^12,24,35^. Indeed, most of the earthquakes which are associated with positive changes of the water level occur in normal faulting or strike-slip regimes. Examples are the Koyna-Warna reservoirs in India^14,24,36^ and the Aswan Dam in Egypt, Africa^37^, respectively. In contrast, events associated with reservoir impoundment located in thrust faulting tectonic regions correlate with unloading of the reservoir, e.g. the Tarbela dam in Pakistan^38^.

### The Conceptual Model of Underground Reservoir-Induced Seismicity

Previous studies demonstrated that seismogenic processes in the study area are rather complex, leading to a debate on governing physical mechanisms^8,11^.

The model approach developed in this work is based upon the concept of RIS (e.g.^12,24^), motivated by the following observations: Firstly, due to the slightly underpressured Arbuckle aquifer^39^, waste water in the study area is usually disposed by gravity^30^. In contrast, injection pressures at Enhanced Geothermal Systems or for shale gas production by hydraulic fracturing amount to multiples of the *in situ* formation pressure. Secondly, unlike the rather local effect of high-pressure fluid stimulations, numerous disposal wells cover the study area which includes also large fault zones^29,30,40^. Thirdly, disposed cumulative fluid volumes in Central Oklahoma were reported to be as high as 200 million cubic meter^3^, i.e. much larger than those injected for fluid stimulations. Lastly, seismicity also occurs at large distances not directly connected to single injectors^8^. The same observation has been noted in previous studies on RIS, where the event locations are remote from the water column (see e.g.^34^). Thus, poroelastic coupling effects might play an important role.

**Figure 2:**
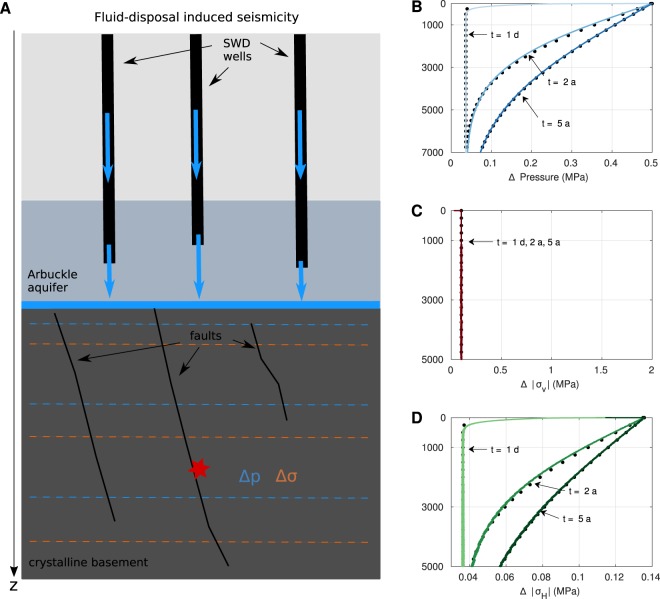
The conceptual model of underground reservoir-induced seismicity (URIS) and pressure and stress solutions. (**A**) Salt water disposal (SWD) wells (black bars) inject into the highly permeable Arbuckle aquifer (grey-blue square). As the basement has a low permeability, the injected fluid forms an effective layer of height Δ*h* (blue layer) on top of the basement (dark grey). Such a scenario can be considered as the filling of an underground reservoir. Caused by the weight of the water column and the pore-fluid pressure beneath Δ*h*, stress- and pressure changes penetrate the poroelastic basement (dashed orange and blue lines, respectively). The perturbation of the ambient stress state may cause shear failure of pre-existing, optimally oriented, critically stressed faults. Changes of pressure (**B**) and stress (**C**,**D**) as profiles along the depth for times 24 h, 2 a and 5 a (colour-coded from light to dark) obtained from the analytic solution (black dots) and FEM (solid lines) for a constant boundary condition *p*_0_. Pore-fluid pressure profiles are marked by the distinct diffusion-like shape of the profile. With increasing time, the pressure in the medium increases. The maximum principal stress *σ*_1_ acts in vertical direction and is constant with time. In contrast, the minimum principal stress *σ*_3_ is horizontally oriented and time-dependent.

On this basis, our conceptual model deviates from the classic fluid injection scenario such as for geothermal exploration or hydraulic fracturing for shale gas production. We used the knowledge of RIS to assess seismicity patterns in Central Oklahoma. Adapting this concept to the case of waste water disposal, we call the model *underground reservoir-induced seismicity* (URIS), shown in Fig. 2A.

The fluid injected into the underpressured, highly porous and very permeable Arbuckle formation creates an additional pore-fluid pressure as well as an additional vertical elastic (confining) stress. As injection rates were as high as 13 × 10^6^ m^3^ per month in Central Oklahoma^3^ and the crystalline basement has a low permeability, the fluid is expected to form a layer of a certain height Δ*h* on top of the basement in a depth *z*_0_. Note that an exact location of the fluid above the basement top has no significance in terms of the basement loading. Thus, we refer to this situation as an *effective* fluid layer. Such a layer with a higher averaged water column is formed along the seismogenic area overlain by the Arbuckle aquifer. However, we note that because of the extremely high permeability of this formation, this effective water layer is not necessarily concentrated in vicinity to the injection borehole. Returning to the case of RIS, the layer is comparable to the water level in a surface reservoir (artificial lake).

Accepting that the underlying basement acts as a poroelastic half-space, it is a combination of physical mechanisms that induce pressure- and stress changes of the ambient pore-fluid pressure- and stress state in the crystalline. These are the direct effect of mass added to the injection formation and poroelastic coupling which lead to an instantaneous increase of the pore-fluid pressure in the basement, as well as pore-fluid pressure diffusion in the crystalline basement, provoking a delayed increase in pore-fluid pressure.

With the above condition, the 1D poroelastic formulation is applicable^16,17,19,21,25,26^. Modifying equations (2.186)–(2.188) of^26^ for gravity which acts in the vertical *z*-direction, we derive analytical stress and pressure solutions for a constant boundary condition *p*_0_ (see Methods’ Section). In addition to the analytic solution, we developed a finite element model (FEM, see Supplementary Materials and Fig. S4) verified by the analytical pore-fluid pressure and stress solutions.

### Application of URIS to Central Oklahoma

We applied the URIS model to the study area using hydraulic and elastic parameters from literature^10,41^, knowledge of the local geology^27^ and reported data from waste water disposal^3^.

Figure 2B–D, shows computed pressure and stress changes (Δ*p* and Δ*σ*). As the analytic and numerical values coincide, the FEM serves as a basis for future studies. Δ*p* (B) is characterised by two effects: The instantaneous response of the elastic, undrained medium causes the constant value which is approached at greater depth. This effect is superimposed by the delayed response due to pore-fluid pressure diffusion which yields the distinct time-dependent shape of the profiles. The vertical stress perturbation Δ*σ*_*z*_ (C) is constant in time and depth with magnitude *p*_0_, whereas the horizontal stress perturbation Δ*σ*_*x*_ (D) is time- and depth-dependent.

### The Influence of the Tectonic Setting

**Figure 3:**
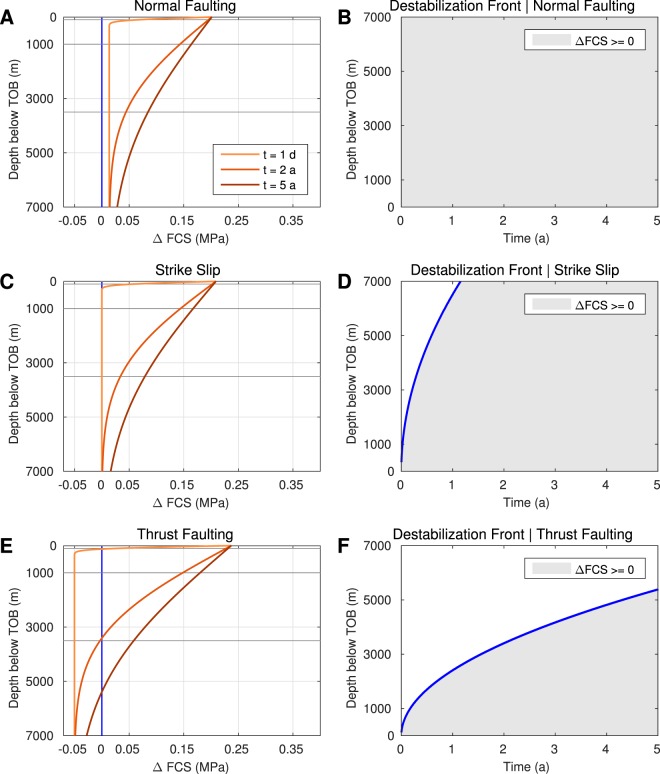
Failure criterion stress and the destabilisation front for different tectonic settings. Based on the analytic solution, Δ*FCS* is computed for a normal faulting (**A**), strike-slip (**C**) and thrust faulting (**E**) regime. The components of Δ*FCS* (orange) are illustrated for times 24 h, 2 a and 5 a (colour-coded from light to dark). While the medium is stable for ${\rm{\Delta }}FCS < 0$, pre-existing, optimally oriented fractures are destabilised if Δ*FCS* turns positive. The location of this transition point can also be expressed in terms of the destabilisation front (blue line, (**B**,**D**) and (**F**).

We used analytic pressure and stress solutions to calculate Δ*FCS* for different tectonic settings (Fig. 3 and Supplementary Materials). Additionally, we introduced the destabilisation front as a measure for the spatio-temporal evolution of the medium destabilisation. In a normal faulting regime, the whole domain is brought closer to failure immediately (Fig. 3A,B), whereas the front migration is strongly delayed for thrust faulting (Fig. 3E,F). In a strike-slip regime (e.g. in Oklahoma) the destabilisation evolves quickly from to TOB to greater depths (Fig. 3C,D).

### Sensitivity to Parameters

The model described above depends on a number of hydraulic and elastic parameters which might be subject to large uncertainties. Aiming at evaluating the model outcome in terms of the parameter choice, we performed a sensitivity analysis, known as ‘one-at-a-time’ (OAT)^42^ (see also Supplementary Materials).

The OAT was run for six hydraulic and elastic parameters which govern the analytic equations (equations 12 to 15). These are the hydraulic diffusivity of the basement *D*, the porosities of the injection formation Φ_*Ar*_ and the basement Φ, the bulk modulus of the pore fluid *K*_*f*_, the P-wave modulus of the drained rock matrix *P*_*mod*,*dr*_ = *λ*_*dr*_ + 2*G*_*dr*_ and the parameter Γ = *n*_*S*_/(*SG*_*dr*_), which characterises the strength of the poroelastic coupling.

**Figure 4:**
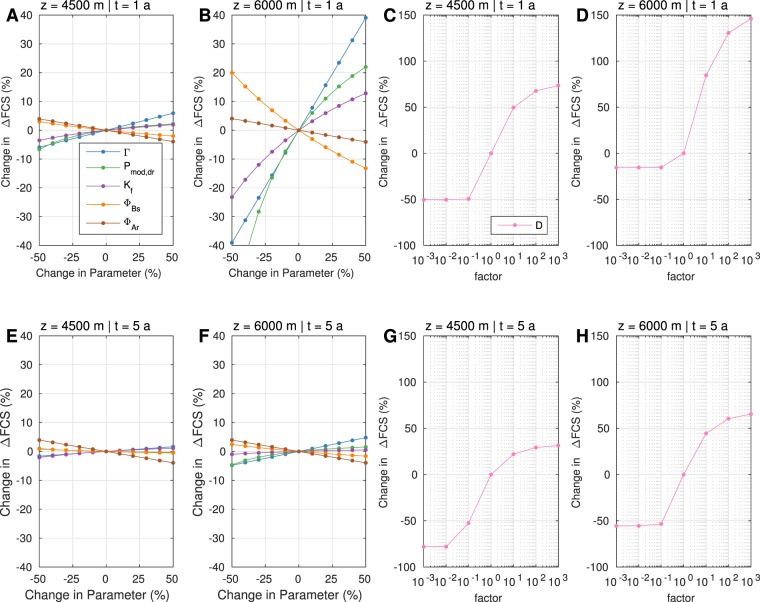
Deterministic sensitivity analysis of input parameters for a strike-slip regime. To account for parameter uncertainties, a sensitivity analysis was performed for hydraulic and elastic parameters Γ, *P*_*mod*,*dr*_, *K*_*f*_, Φ_*Bs*_ and Φ_*Ar*_ (coloured lines, **A**,**B**,**E**,**F**), perturbed incrementally between ±50% while holding the other values at their base values. In contrast, the hydraulic diffusivity *D* (**C**,**D**,**G**,**H**) was varied logarithmically between [1e-3 and 1e3] times the base value. The panels show classical sensitivity plots for 1.5 km and 3 km below the TOB at times 1 a and 5 a (top and bottom). The influence on Δ*FCS* is studied relatively to the base level outcome. In the Supplementary Materials, we additionally demonstrate the influence of the boundary pressure *p*_0_ for different tectonic regimes.

As demonstrated in Fig. 4, Δ*FCS* increases most significantly with *D*, Γ and *P*_*mod*,*dr*_ and decreases strongly for higher Φ. Yet, the dependence is also controlled by the time and location. For example the impact on Δ*FCS* by Γ intensifies with depth, indicating that poroelastic coupling is the controlling mechanism at larger depths. The parameter dependence is also revealed by the destabilisation front. Its spatio-temporal evolution is controlled by Δ*FCS* and thus, by the parameters described above (see Supplementary Materials, Fig. S6).

As known from cases of RIS, the interplay between the water level and the stress regime contributes to the occurrence of seismicity. An additional study on the influence of *p*_0_ in different tectonic regimes (Supplementary Materials) indicates that negative *p*_0_, i.e. fluid discharge from the overlying permeable formation, stabilises the underlying low-permeable medium in a strike-slip regime (Figs S7, A,B and S8, red line) but brings this medium closer to failure in a thrust faulting regime (Figs S7, E,F and S8, green line). The latter observation has also been made for RIS^38^ and implies the importance of considering poroelastic coupling in the URIS model.

### Time-dependent Boundary Condition

Barbour *et al*.^11^ showed that varying injection rates significantly influence poroelastic effects. Since URIS is based on the assumption of an effective fluid accumulation on top of the basement which depends on the injection rates, we defined a time-dependent boundary condition *p*_0_ = *p*_0_(*t*) at the TOB to complement our study.

**Figure 5:**
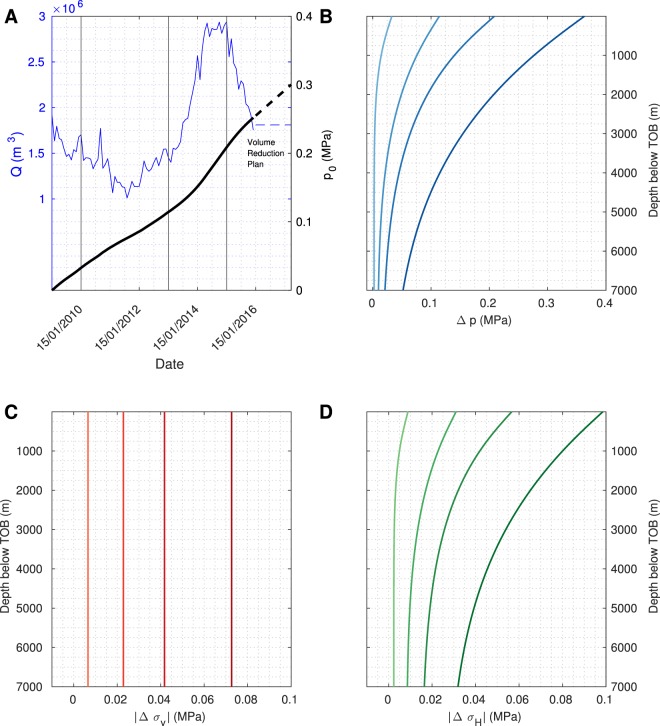
Analytic pressure and stress solutions for a time-dependent boundary condition. To study the influence of the time that the fluid needs to accumulate at the TOB, a time-dependent boundary condition for *p*_0_ is defined, based on the injection rate in our study area between 01/2009 and 12/2015 (blue line, panel (**A**)). We extrapolated the data until 12/2018, using information of the Volume Reduction Plan, to allow for predictions of the seismicity rate after 12/2015. Calculating ${\int }_{0}^{t}\,Q(\tau )\,{\rm{d}}\tau$, yields *p*_0_(*t*). Solving analytically for equations (2)–(5) and subtracting the gravity effect yields changes of the pore-fluid pressure (**B**), the vertical (**C**) as well as the horizontal (**D**) stress. Colour-coding shows the corresponding profile along the depth for times 01/2010, 01/2013, 01/2015 and 12/2018 (light to dark colours). In spite of the fact that the pressure perturbation is generally higher than stress changes, at some depths and times they can have the same order of magnitude. This results in various signatures of URIS under different stress regimes and is a direct result of the poroelastic coupling.

The evolution of *p*_0_(*t*) (Fig. 5A, black line) is computed using the monthly injected fluid volume *Q*(*t*) in our study area^3^ (Fig. 5A, blue line). After the triggering threshold had been reached in 2009, seismicity rates in Central Oklahoma significantly increased. Thus, we assume that fluid began to effectively accumulate on top of the basement by that time (model time *t* = 0 a). Since injection rates are not given for times after 15 Dec 2015, we extrapolate for dates until 15 Dec 2018 on a constant value based on the Volume Reduction Plan (see Introduction section). Calculating ${\int }_{0}^{t}\,Q(\tau )\,{\rm{d}}\tau$ yields the magnitude of *p*_0_(*t*) divided by a factor Ω. Here, Ω depends on the size of the study area, the fluid density and the gravitational acceleration constant.

Using *p*_0_(*t*), we solved for the analytical pressure and stress equations 12–15 (Fig. 5B–D). As for the constant boundary condition, the change of the vertical principal stress is independent of the depth but as *p*_0_ changes with time, this stress becomes a function of time.

### Synthetic Seismicity versus Central Oklahoma Seismicity

Increasing shear stress, decreasing normal stress, as well as a reduction of the effective normal stress due to higher pore-fluid pressure can be assumed to increase the failure stress *FCS*. If these changes are larger than a critical threshold value, a seismic event is triggered^26,43^.

Based on that idea, we apply the triggering criterion introduced by Rothert and Shapiro^44^: Δ*FCS*(*z*,*t*) ≥ *C*(*z*) for the generation of synthetic events. Here, Δ*FCS* is the change in failure criterion stress, obtained from the analytical solution. The quantity *C*(*z*) characterises the strength of pre-existing fractures and faults (criticality) and is governed by rock parameters and the tectonic setting. Seismicity can only happen at locations where the criticality is low, meaning at locations close to failure. At each time step, the value of Δ*FCS* is compared to the local critical value *C*(*z*). If the triggering criterion is full-filled, a seismic event is triggered and the critical value is set to infinity to exclude multiple triggering at one location.

**Figure 6:**
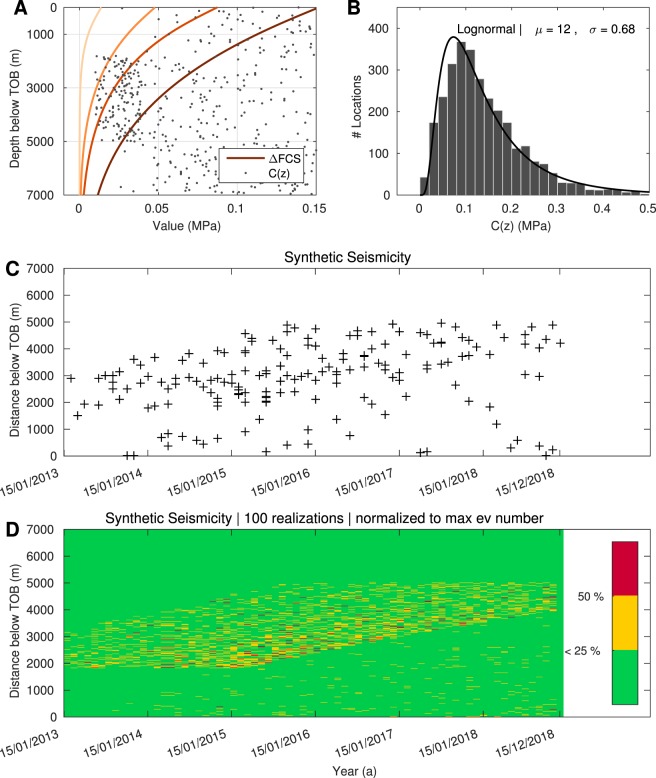
Generation of synthetic seismicity. Accepting that Central Oklahoma is characterised by strike-slip conditions, Δ*FCS* is calculated (**A**) using analytical pressure and stress solutions for a time-dependent *p*_0_ (Fig. 5). The triggering criterion ${\rm{\Delta }}FCS(z,t)\, > \,C(z)$ can then be used for the generation of synthetic events. Here, *C*(*z*) is the log-normally and heterogeneously distributed criticality (**B**). An *r*−*t*-plot of the event cloud (**C**) for the interval 01/2013 to 12/2018 illustrates that the farthest events occur 5000 m below the TOB. This result is stable, evaluating 100 random realisations of *C*(*z*) (**D**). Regions which are most likely to fail are marked in red (locations failed in >50% of the 100 runs).

For the computation of Δ*FCS*, recall that we set Δ*σ*_*H*_ = Δ*σ*_*h*_, justified by the plane strain model (see Supplementary Materials). Thus, in a strike-slip regime Δ*σ*_*m*_ = Δ*σ*_*h*_ and Δ*σ*_*d*_ = 0 (shown in Fig. S9). Figure 6A shows profiles of Δ*FCS* along the depth below the TOB for the dates 15/01/2010, 15/01/2013, 15/01/2015 and 15/12/2018. Driven by the diffusion of pore-fluid pressure, destabilisation of the medium evolves with time from the TOB to the deeper basement. The constant value of *p* reached at greater depth can be attributed to the elastic response of the medium due to poroelastic coupling effects.

The criticality field *C*(*z*) is calibrated on the basis of the spatio-temporal evolution of events in Central Oklahoma as well as on geological features in the study area. This is a first step necessary for the modelling. Accepting that faults close to failure exist in the basement of Central Oklahoma^29^, we define the magnitude of *C* by log-normally distributed statistically random numbers which means comparatively more low critical stress values (see Methods’ section). Moreover, seismicity in the study area neither occurs in the injection formation (Arbuckle aquifer), nor in the top layers of the basement. Thus, further assumptions have to be made. It has been noted that especially larger magnitude earthquakes happened in zones of increased P-wave velocity, corresponding to tectonically weak areas. On the contrary, hardly any seismicity is observed in regions of low *v*_*p*_/*v*_*s*_ ratios which exist in the upper basement^40^. Therefore, we define a number of locations with a relatively smaller value of *C*(*z*) at depths between 2 km and 5 km below the TOB as well as higher values in the upper basement (small values of *C*(*z*) mean that locations are closer to failure). Overall, the statistics of *C*(*z*) still follow a log-normal distribution (see Fig. 6B).

Spatio-temporal characteristics of the simulated events are graphically presented in an *r*−*t*-plot (Fig. 6C) for the time 01/2013 to 12/2018. Whereas the main activity occurs between 2 km to 5 km below the TOB, rather little activity is observed close to the basement top. Taking into account 100 realisations of *C*(*z*), this result is confirmed to be stable (Fig. 6D). At the time of the burst in seismicity in 2013, locations most prone to failure (yellow and red) occurred in depths of up to 3 km below the TOB. With time, the seismogenic zone shifts to greater depths but not deeper than 5 km below the TOB (Fig. S7B).

These spatio-temporal features are in general agreement with the analysed Central Oklahoma main shocks observed between mid-2013 and early 2016 (Figs 1 and S7A): Between 2013 and 2016, the seismogenic zone extends between 2–5 km below the TOB, whereas rather little activity is observed close to and deeper than 5 km below the TOB. Furthermore, the maximum event depth migrated from 3 km to 5 km below the TOB and the mean event depth shifted from 2.5 km to 4 km below the TOB.

**Figure 7:**
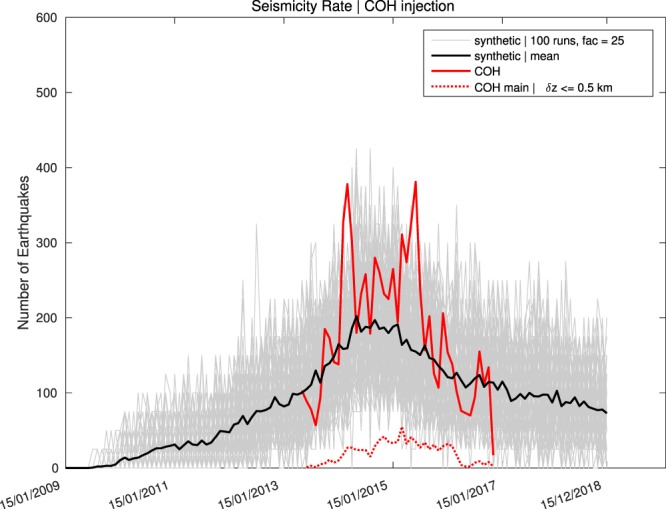
Seismicity Rates for observed and synthetic events. An important feature of earthquakes induced in Oklahoma is the seismicity rate. Using the whole catalogue for the study area Central Oklahoma (COH)^29^, the event number started to increase in 2013 and decayed in early 2016. Calculated synthetic rates for 100 realisations of *C*(*z*) (grey lines) resolves the observed temporal behaviour (the mean is marked by the bold black line) and thus, should be considered as suitable for predictions on the seismic response to varying injection rates.

Regarding seismicity rates we do not depend on high-precision localised events. Thus, in the following, we focus on the complete catalogue of relocated events in the study area. As shown in Fig. 7, seismic activity significantly increased in 2013 and started to decay in late 2015, continuing at least until the time of the last event comprised in the data in November 2016 (compare to Fig. 1 in Langenbruch and Zoback^45^). Comparing this trend to synthetic event rates, we find that our model is supported by the observation of decreasing rates throughout 2016. Using the predictive nature of our model until 15/12/2018, we find that the declining number of events persists throughout that interval. This observation is in agreement with a recent study by Langenbruch and Zoback^3,45^.

## Discussion

We extended the model of RIS caused by the filling of artificial lakes to seismicity induced by high-volume underground fluid disposals, URIS.

Overall, the results show that URIS preferably occurs in normal faulting or strike-slip tectonic regimes and is time-delayed in thrust faulting. That is a noteworthy difference from RIS. The sensitivity study demonstrated that the consideration of poroelastic coupling plays an important role for URIS. In a strike-slip regime, the poroelastic effect controls the change of the failure stress at greater depths and early times.

For the application of the URIS model to Central Oklahoma, we defined a time-dependent boundary condition *p*_0_(*t*) based on reported values for the monthly injected fluid volume in the study area between 15/01/2009 and 15/12/2015. To include the possibility of predictions for the time after 2015, we extrapolated the injection rate until 12/2018 to a constant value following from the Volume Reduction Plan of OCC (i.e. a 40% reduction of the total volume injected in 2014).

Using the obtained values of the change in failure criterion stress for a strike-slip tectonic setting, a catalogue of synthetic events was generated. Whereas little activity occurs close to the top of the basement, a larger number of events is observed between 2 and 4 km below the basement top. These larger event depths might be attributable to the instantaneous elastic response of the rock matrix. With time, the mean event depth migrates to greater depths which can be explained by the time-dependent pore-fluid pressure diffusion. Overall, the evolution of events in time and depth coincides with the event distribution observed in Central Oklahoma.

Our findings demonstrate that the presented novel physical model of URIS captures spatio-temporal features of the seismicity that was observed in Central Oklahoma within the last decade. Additionally, we demonstrated that our model resolves well the seismic response to the injection rate in the area of interest. Therefore the approach has a predictive power for the seismicity rate even in case of decaying fluid disposal rates.

To conclude, the work presented here will help in understanding controlling physical processes related to high-volume fluid injections such as waste water disposal. This is of importance for hazard assessment and seismic risk mitigation not only in Oklahoma but globally.

### Model Limitations

We note that the model developed here comprises only principal features of the phenomena.

First, regarding the spatio-temporal evolution of seismicity linked to waste water injections, it is the interaction of various parameters that controls the magnitude and number of events. Not only does this include the magnitude and orientation of pre-existing stresses, but also the orientation of faults, the injection trajectory^12^ as well as hydraulic and elastic parameters. The influence of parameters on Δ*FCS* was investigated in a sensitivity study. At this point, the application of OAT to the pressure- and stress solutions is a first step before implementing more time-consuming global sensitivity analyses combined with a 3-D numerical model and Monte Carlo simulations. Accepting the destabilisation front as a measure for the spatio-temporal seismicity evolution, it should be noted that the event distribution is indeed influenced by the parameters used in this study. Yet, within parameter variations of +/− 10%, the location of the front is still within the range of event location uncertainties.

Second, the injection scenario in the area of interest is rather complex. We do not compute exact boundary pressures and stresses at the TOB for each injector but rather consider a cumulative volume for all injectors in Central Oklahoma. Additionally, the assumption of a constant injection rate after 12/2015 evokes uncertainty in the predictive nature of our modelling results after 12/2015.

Third, the spatio-temporal evolution of synthetic events is clearly influenced by the definition of the criticality field *C*(*z*). However, the choice of values is justified by the distribution of observed earthquakes as well as geologic features in the study area. Performing P wave tomography in Oklahoma, Pei *et al*.^40^ suggest that seismicity in that area tends to occur in high-velocity regions which can be found in the deeper basement. In contrast, the upper basement is characterised by low *v*_*p*_/*v*_*s*_ ratios. Thus, geological structures seem to control earthquake locations which justifies the choice of a heterogeneous distribution of the criticality field *C*(*z*).

Last, we do not account for subsurface structures which may lead to local stress accumulations and increased pressure perturbations^46^. Moreover, large fault zones act as fluid flow channels, causing a rapid response of deeper basement intervals.

Nevertheless, we successfully applied the new model of URIS to the case of earthquakes observed in Central Oklahoma. Thus, it provides an important step towards future research on the characterisation and analysis of seismicity induced by high-volume fluid injections.

## Methods

### Data

We use locations and occurrence times of seismic events from a catalogue published by Schoenball and Ellsworth^29^. Given depths are referred to the ground surface. To analyse only the spatio-temporal distribution of main shocks, we declustered the catalogue (see the Supplementary Materials).

The top of the basement (TOB) was constrained by well data, obtained from Campbell and Weber^31^. We plot here the surface of the basement top relative to the ground surface elevation. Based on this data, we set the mean depth of the TOB *z*_0_ to 3 km for our calculations.

Injection rates for the study area were taken from Langenbruch and Zoback^3^.

### Analytic Solution

For the derivation of the analytical solution, we assume that a medium with porosity Φ_*Ar*_ overlays a fluid-saturated, poroelastic half-space which extends in the vertical *z*-direction below the depth *z*_0_. In such a case, the application of a surface load on the poroelastic half-space can be described by a 1D problem and quantities are functions of *z* only. The stress-strain relation:

2 $${\sigma }_{ij}+\alpha p{\delta }_{ij}=2G{\varepsilon }_{ij}+\frac{2G{\rm{\nu }}}{1-2{\rm{\nu }}}\varepsilon {\delta }_{ij}\,$$

for such an uniaxial-strain problem is given by^19,26,47^:

3 $${\sigma }_{zz}=({\lambda }_{dr}+2{G}_{dr}){\varepsilon }_{zz}-\alpha p$$

4 $${\sigma }_{xx}={\sigma }_{yy}={\lambda }_{dr}{\varepsilon }_{zz}-\alpha p\,\mathrm{.}$$

In the above equations, *σ* and *ε* are the stress and strain, respectively. *p* denotes the pore-fluid pressure and *α* is the so-called Biot’s coefficient. Furthermore, *λ*_*dr*_ and *G*_*dr*_ are the drained Lamé parameters. Equations 3–4 are valid under the assumption of pore space being filled with a fluid with pressure *p*. Then, the pore-fluid pressure relation:

5 $$p=M(\zeta -\alpha {\varepsilon }_{kk})\,$$

for the uniaxial strain case can be written as (equation (2.176) in Shapiro^26^):

6 $$p=M(\zeta -\alpha {\varepsilon }_{zz})\,\mathrm{.}$$

Here, *ζ* is the relative modification of pore-space volume due to fluid mass changes and *M* is the Biot’s modulus $M={[\frac{{\rm{\Phi }}}{{K}_{f}}+\frac{\alpha -{\rm{\Phi }}}{{K}_{dr}}]}^{-1}$, with the porosity Φ, the Biot’s coefficient *α* and the bulk moduli of the drained matrix *K*_*dr*_ and the pore fluid *K*_*f*_.

The equilibrium equation for poroelastic stresses, assuming that gravity acts in the vertical *z*-direction, is given by:

7 $$\frac{\partial }{\partial z}{\sigma }_{zz}=-\,\rho g\,,$$

with the bulk density *ρ* = *ρ*_*dr*_ + Φ*ρ*_*f*_, where *ρ*_*f*_ and *ρ*_*dr*_ denote the fluid density and the drained matrix density of a medium with porosity Φ, respectively. Following the concept of loading modes introduced by Detournay and Cheng^19^, field quantities can be obtained. All of the above hydraulic and elastic parameters are values for the underlying formation (the crystalline basement in Central Oklahoma).

Loading mode I corresponds to Terzaghi’s 1D consolidation. In this mode, a normal confining stress of magnitude Φ_*Ar*_*p*_0_ acts at the top boundary of the poroelastic half-space at depth *z*_0_ and pore-fluid pressure perturbation on the surface *z*_0_ is absent. We introduced the porosity of the target injection formation Φ_*Ar*_, in our case the Arbuckle formation. Note that this is a modification of the 1D solution as described by Shapiro^26^ and an important feature of the URIS model. The boundary condition for the stress at *z*_0_ is then given by ${\sigma }_{zz}^{I}({z}_{0},t)=-\,{H}_{s}-h(t){{\rm{\Phi }}}_{Ar}{p}_{0}$. *h*(*t*) indicates the step-like increase of stress at time *t* = 0 and *H*_*s*_ is a constant corresponding to the load caused by the overburden. Using the equilibrium equation (7), the total stress is a function of the depth and given by:

8 $${\sigma }_{zz}^{I}(z,t)=-\,{H}_{s}-\rho g(z-{z}_{0})-h(t){p}_{0}{{\rm{\Phi }}}_{Ar}\,\mathrm{.}$$

Immediately after the load is applied, the system is in an undrained state and fluid flow is vanishing. From equation (2.179) of^26^ it follows:

9 $$p(z,t)={p}_{0}{\rm{erfc}}(\frac{z}{\sqrt{4Dt}})\,\mathrm{.}$$

Using this relation, the pore-fluid pressure in loading mode I can be calculated by:

10 $${p}^{I}(z,t)={\rho }_{f}g(z-{z}_{0})+{{\rm{\Phi }}}_{Ar}\frac{{n}_{S}}{{G}_{dr}S}[{p}_{0}h(t)-{p}_{0}{\rm{erfc}}(\frac{z-{z}_{0}}{\sqrt{4Dt}})]\,,$$

(see also equation (2.184) of^26^). *S* and *D* are the uniaxial storage coefficient and the hydraulic diffusivity, respectively, and *n*_*S*_ is the poroelastic stress coefficient *n*_*S*_ ≡ *α*(1 − 2*ν*_*dr*_)/2(1 − *ν*_*dr*_) with the drained Poisson’s ratio *ν*_*dr*_.

In loading mode II, a pore-fluid pressure *p*^*II*^(*z*_0_,*t*) = *H*_*f*_ + *p*_0_*h*(*t*) is applied at *z*_0_ and normal stresses are vanishing. In analogy to the constant load induced by the overburden *H*_*s*_, *H*_*f*_ is a constant corresponding to the load caused by the fluid in the pore space of the overburden. In our scenario, *H*_*f*_ is given by the *in-situ* pore-fluid pressure *H*_*f*_ = *ρ*_*f*_ *gz*_0_. Thus, it follows:

11 $${p}^{II}(z,t)={H}_{f}+{p}_{0}{\rm{erfc}}(\frac{z-{z}_{0}}{\sqrt{4Dt}})\,\mathrm{.}$$

The complete solution to this boundary problem is than obtained by a summation of the pressure and stress solutions for modes I and II:

12 $$p(z,t)={H}_{f}+{\rho }_{f}g(z-{z}_{0})+{p}_{0}h(t)[{{\rm{\Phi }}}_{Ar}\frac{{n}_{S}}{{G}_{dr}S}+{\rm{erfc}}(\frac{z-{z}_{0}}{\sqrt{4Dt}})[1-{{\rm{\Phi }}}_{Ar}\frac{{n}_{S}}{{G}_{dr}S}]]\,,$$

13 $${\sigma }_{zz}(z,t)=-\,{H}_{s}-\rho g(z-{z}_{0})-h(t){p}_{0}{{\rm{\Phi }}}_{Ar}\,\mathrm{.}$$

Further it follows for the vertical strain *ε*_*zz*_(*z*,*t*) and horizontal stress *σ*_*xx*_(*z*,*t*):

14 $${\varepsilon }_{zz}(z,t)=\frac{{\sigma }_{zz}(z,t)+\alpha p(z,t)}{{\lambda }_{dr}+2{G}_{dr}}\,,$$

15 $${\sigma }_{xx}(z,t)={\sigma }_{yy}(z,t)={\lambda }_{dr}{\varepsilon }_{zz}(z,t)-\alpha p(z,t)\,\mathrm{.}$$

The multiplication with *h*(*t*) yields a step-like increase of *p*_0_ at time *t* = 0 to a constant value *p*_0_. Later we also show results for a time-dependent boundary condition where *p*_0_ = *p*_0_(*t*). Note that a multiplication of ${p}_{0}{\rm{erfc}}(\frac{z-{z}_{0}}{\sqrt{4Dt}})$ with *h*(*t*) does not change the solution (see also equation (2.186) in^26^).

For pressure and stress calculations, we use *z* = [0, 10 km] with a spacing of 1 m.

### Numerical Model

The COMSOL Multiphysics software applied for the solution of the numerical model is a finite element software. For our purposes we use version 5.2a and the built-in Poroelasticity interface, which couples the Fluid Flow and Solid Mechanics physics (see Supplementary Materials).

Prior to further analyses, the numerical pressure and stress values obtained for the model geometry of 1 m length and total depth of 30 km were interpolated on a regular two-dimensional grid with a spacing of 0.1 m in *x*-direction and 1 m in *z*-direction. Subsequently, we extracted values which lie on a line which extends in z-direction between [*z*_0_, 10 km] at *x* = 0.5 m.

### Synthetic Seismicity

The log-normal distribution is given by the probability density function y = $f(x|\mu ,\sigma )=\frac{1}{x\sigma \sqrt{2\pi }}{e}^{\frac{-{(lnx-\mu )}^{2}}{2{\sigma }^{2}}}$, where $\mu =\,\mathrm{ln}({m}^{2}/\sqrt{v+{m}^{2}})$ and $\sigma =\sqrt{\mathrm{ln}(v/{m}^{2}+\mathrm{1)}}$ are parameters of the distribution with the mean *m* and the variance *v*. We find that the spatio-temporal distribution of synthetic events and observed seismicity at different depth intervals is most consistent for a criticality field statistically defined by a log-normal distribution with *m* = 0.15 MPa and *v* = 10 GPa^2^. Additionally, zones in the upper basement (above 2 km) and lower basement (below 2 km) are characterised by uniformly distributed values between [0.1 MPa, 0.3 MPa] and [0.01 MPa, 0.4 MPa], respectively. The locations *z* of *C*(*z*) are given by the same spatial grid as used for the analytical solution, i.e. *N* points along a line which extends in *z*-direction between *z*_0_ = 3 km and 10 km with a grid spacing of 1 m.

### Data Availability

The synthetic datasets generated and analysed during the current study are available from the corresponding author on reasonable request. Well data used to constrain the basement depth are available from, http://ogs.ou.edu/docs/specialpublications/SP2006-1T1.xls. The catalogue of relocated events used in this work can be obtained from^29^, Supplementary Material.

## Electronic supplementary material

Supplementary Materials
