# Supplementary material for: The surge of earthquakes in Central Oklahoma has features of reservoir-induced seismicity
Source: Sci Rep. 2018 Jul 31;8:11505. doi: 10.1038/s41598-018-29883-9 (PMC6068131; doi:10.1038/s41598-018-29883-9)
Supplement: Supplementary file 1 — Supplementary Materials [file 41598_2018_29883_MOESM1_ESM.pdf]

The surge of earthquakes in Central Oklahoma has features of reservoir-induced seismicity

Lisa Johann[1,*], Serge A. Shapiro[1], and Carsten Dinske[1]

[1]Freie Universitaet Berlin, Institute of Geophysics, Berlin, 12249, Germany

[*]lisa.johann@geophysik.fu-berlin.de

[+]these authors contributed equally to this work

ABSTRACT

This file contains supplementary material for the main article, necessary to comprehend the background of the study and the methods we applied.

Supplementary Material includes

- Supplementary Text
- Fig. S1 – S10
- Table S1
- References

Supplementary Text

S1. Declustering

We use locations and occurrence times of seismic events from a catalogue published by Schoenball and Ellsworth[1].

As demonstrated in previous works, naturally occurring earthquakes are distributed according to a non-homogeneous Poisson process (NHPP) in time, reflecting the existence of fore- and aftershock sequences[2]. In contrast, a sequence of independently occurring events can be statistically described by a homogeneous Poisson process (HPP).

Recently published works revealed that seismicity in Oklahoma shows significant aftershock activity[3]. Thus, for the work presented here, we removed these dependent events from the original earthquake catalogue[1], neglecting events with a depth error $\delta z > 0.5$ km (Fig. S1. We applied a declustering method by Urhammer[4] which is based on a moving time and space window. From 7835 events located in our area of interest, 2473 events had a vertical error $\zeta < 0.5$ km. Among these high-precision events, 1263 main shocks were identified by the algorithm (Fig. S2).

As shown in Figure S3, A - B, the main shocks (red line) now follow the expected homogeneous Poisson distribution (HPP), demonstrated by a simulated event distribution (black dashed line). To describe the magnitude-frequency distribution, the Gutenberg-Richter relation[5] given by $\log_{10} N = a - bM$ is used. Here, N is the number of events with magnitude $\geq M$ and a and b are constants. For the declustered event catalogue, $b = 1.11$ (Fig. S3C). The obtained magnitude of completeness is $M_c = 2.4$.

As can be seen in Fig. S10A, the mean depth of the main shocks with $M > M_c$ increases with time.

S2. Numerics

The COMSOL Multiphysics software applied for the solution of the numerical model is a finite element software. For our purposes we use version 5.2a and the built-in Poroelasticity interface, which couples the Fluid Flow and Solid Mechanics physics

Equations The numerical solutions are based on the quasi-static approximation of poroelasticity. This

approximation is given by the continuity equation:

$$\frac{\partial \Phi}{\partial t} + \nabla \mathbf{u} = 0\,, \tag{S1}$$

defining the conservation of fluid mass, Darcy's law:

$$\mathbf{u} = -\frac{\kappa}{\eta}\nabla p\,, \tag{S2}$$

and the equilibrium equation for poroelastic stresses:

$$\nabla \sigma = 0\,. \tag{S3}$$

Here, κ denotes the permeability and η is the dynamic viscosity of the pore fluid. Equations S1 - S2 are valid for a hydraulically and elastically isotropic, homogeneous medium.

Using the two constitutive equations (1) and (4) with equations S1 - S2, solutions for pore-fluid pressure and stresses can be found. There exist several equivalent forms of pressure and stress equations in literature (see e.g. chapter 2.5 in[6]). Assuming a hydraulically and elastically isotropic, homogeneous medium, pore-fluid pressure can be obtained by the solution of a diffusion equation coupled to isotropic stress terms using the quantity M, the permeability κ and the fluid viscosity η:

$$\frac{1}{M}\frac{\partial p}{\partial t} - \frac{\kappa}{\eta}\nabla^2 p = -\alpha\frac{\partial \varepsilon_{kk}}{\partial t}\,. \tag{S4}$$

In this equation, the right-hand term can be interpreted as the rate of change of pore space. As increasing $\partial\varepsilon_{kk}/\partial t$ gives rise to a larger volume available for fluid storage, it indicates a fluid sink marked by the negative sign.

Stresses are obtained from the equilibrium equation S3, assuming that the solid is under purely gravitational load:

$$\nabla \sigma = -\left(\rho_f \Phi + \rho_{dr}\right)\mathbf{g}\,. \tag{S5}$$

Even though equation S5 is a stationary expression, it is applicable for time-dependent flow models as in our case. This is because the elastic response of the undrained medium is generally much faster than the fluid flow. Thus, in response to changing flow conditions, a new stress equilibrium is reached immediately and stresses and strains become functions of time. Such an assumption is considered as the quasi-static approximation of poroelasticity.

Geometry

We build our approximate model using a 2D plane strain geometry, assuming that the horizontal length in y-direction is much larger than the other two directions x and z. Thus, perturbations of the normal strain $\Delta\varepsilon_{yy}$ as well as of the shear strains $\Delta\varepsilon_{xy}$ and $\Delta\varepsilon_{zy}$ are zero and the stress perturbations in the horizontal plane are equal: $\Delta\sigma_{yy} = \Delta\sigma_{xx}$.

We define a rectangular geometry with length $x = 1$ m and total depth $z = 30$ km (Fig. S4), representing the subsurface of Oklahoma. The small extension in x-direction is justified by the aim of modelling effectively a 1D problem. We tested the influence of the vertical boundaries to exclude the possibility of distorted results. An additional boundary is inserted at $z = z_0 = 3$ km (Fig. S4.2b, bold line at 3 km). It defines the boundary between the Arbuckle formation and the basement and thus, also the bottom of the reservoir coinciding with the TOB. For the following we assume that the Cartesian coordinate system is oriented such that principal axes align with the x- and z-directions.

Parameter, Boundary and Initial Conditions

For the assessment of seismicity induced in Central Oklahoma, numerical and analytical models are based on hydraulic and elastic parameter from literature, listed in table 1. Note that all parameters are defined for the crystalline basement in Oklahoma except for the porosity of the injection formation Φ_{Ar}. Regarding the Biot's coefficient α, we use a value of 0.3. This is only an estimate as elastic parameters strongly depend on the stress conditions under which they are measured. Also larger values have been reported for granite in literature (see e.g.[7]). The larger α, the stronger the poroelastic coupling. Thus, the comparatively low value can be understood to represent one end member of rather weak poroelastic coupling.

As we consider a poroelastic finite element model, appropriate hydro-mechanical boundary (BC) and initial conditions (IC) have to be set. Regarding mechanical BC, the top of the block defines the

earth's surface and is set to free. The other three boundaries are defined by a so-called roller condition, i.e. no lateral movement. Corresponding hydrological BC are given by no flow at the side and bottom boundary. Regarding the top boundary corresponding to the surface, the pressure is set to $p = 0\,\text{Pa}$. The TOB-boundary at depth z_0 becomes active for times $t \geq 0$. At this boundary, the pressure is given by the pressure at the bottom of the reservoir due to the overlying water column $p(z_0,t) = p_0$ and by the vertical stress $\sigma_{zz}(z_0,t) = -p_0\Phi_{Ar}$.

Concerning IC, we include gravity driven stress- and pressure gradients. For this, two stationary model runs are necessary prior to the transient analysis including the TOB-boundary. In step one, initial displacement u and pressure p are set to zero and the solver calculates ambient stress and pressure values due to gravity driven loading (Fig. S4.1). Step two, referring again to a stationary solver, is needed for the model calibration as well as for software intern definition issues. A second poroelasticity physics module is added and pressure and stress solutions derived in step one are defined as IC (Fig. S4.2a). The third step solves the transient poroelastic equations for times $t = [0,\, t_{max}]$ with $\Delta t = 1$ month. For this, pressure and stress solutions obtained in step two are defined as initial values. Note that the additional boundary defining the bottom of the reservoir only becomes active in this study step (Fig. S4.2b).

Data Analysis

Prior to further analyses, the numerical pressure and stress values obtained for the model geometry of 1 m length and total depth of 30 km were interpolated on a regular two-dimensional grid with a spacing of 0.1 m in x-direction and 1 m in z-direction. Subsequently, we extracted values which lie on a line which extends in z-direction between $[z_0,\, 10\,\text{km}]$ at $x = 0.5\,\text{m}$.

S3. The Influence of the Tectonic Setting

As shown above and in earlier studies on RIS[6,8], the tectonic setting significantly influences the occurrence of reservoir induced seismic activity. These works demonstrated that RIS is most likely under normal faulting and strike-slip stress orientations. Therefore, the ambient state of stress should be considered also in the case of URIS.

To validate this assumption, we use the analytically derived pressure and stress solutions and calculate values of ΔFCS for three arbitrary stress regimes. The initial values are set such that the differential stress

is equal for all cases and that magnitudes are larger than the calculated perturbations. Further, the type of the background state of stress (faulting regime) remains unaffected by the fluid injection. Note that the numerical models were obtained for plane strain solutions, i.e. stresses changes in the horizontal plane are equal ($\Delta\sigma_x = \Delta\sigma_H = \Delta\sigma_y = \Delta\sigma_h$). Under this limitation, total vertical, maximum horizontal and minimum horizontal stresses are given by

$$\sigma_v = \sigma_{v,ini} + \Delta\sigma_z, \tag{S6}$$

$$\sigma_H = \sigma_{H,ini} + \Delta\sigma_x, \tag{S7}$$

$$\sigma_h = \sigma_{h,ini} + \Delta\sigma_x, \tag{S8}$$

respectively.

Since a pre-existing, optimally oriented fracture is stable as long as ΔFCS is negative but is moved closer to failure if ΔFCS turns positive, the time and location of destabilisation can be determined. We call the location $z(t)$ for which $\Delta FCS = 0$ the *destabilisation front*. Earlier works on induced seismicity use so-called $r-t$-plots for the analysis of spatio-temporal features of the seismic cloud[9,10]. In the 1D case considered here, r corresponds to the event depth. Plotting the evolution of the destabilisation front in an $r-t$-plot, this front envelops the domain that has been brought closer to failure ($\Delta FCS > 0$) due to pressure and stress perturbations.

Figure 3 shows the obtained values of ΔFCS for the different regimes as profiles along the depth for different time steps as well as the corresponding destabilisation front. The components of ΔFCS are depicted in Figure S5. Of course, the individual magnitudes must be considered carefully as they strongly depend on the initial conditions as well as on used model parameters.

Normal Faulting

Let us consider a normal faulting regime with

$$\sigma_{1,ini} = \sigma_{v,ini}$$

$$\sigma_{2,ini} = \sigma_{H,ini} = 0.9\sigma_{v,ini}$$

$$\sigma_{3,ini} = \sigma_{h,ini} = 0.75\sigma_{v,ini}$$

Under the assumption that initial stress magnitudes are larger than the calculated perturbations it follows for the principal stresses with equations S6 - S8: $\sigma_1 = \sigma_{v,ini} + \Delta\sigma_z$, $\sigma_2 = \sigma_{H,ini} + \Delta\sigma_x$, $\sigma_3 = \sigma_{h,ini} + \Delta\sigma_x$. From this it follows that $\Delta\sigma_m = 0.5\left(\Delta\sigma_z + \Delta\sigma_x\right)$ and $\Delta\sigma_d = \Delta\sigma_z - \Delta\sigma_x$.

As shown in Figure S5A, the contribution of $-\sin(\varphi)\Delta\sigma_m$ is negative throughout the medium and the model time, indicating positive values of $\Delta\sigma_m$ which leads to a stabilisation of the medium. However, $0.5\Delta\sigma_d$ is smaller than $\sin(\varphi)\left(\Delta\sigma_m - \Delta p\right)$ such that ΔFCS is positive at each location and time step (Fig. 3A). This behaviour is also revealed by the domain of destabilisation, which evolves immediately even at the deepest point of our model (Fig. 3B).

Strike-Slip

Now, let us take a strike-slip regime with

$$\begin{aligned}
\sigma_{1,ini} &= \sigma_{H,ini} = 1.05\sigma_{v,ini} \\
\sigma_{2,ini} &= \sigma_{v,ini} \\
\sigma_{3,ini} &= \sigma_{h,ini} = 0.8\sigma_{v,ini}
\end{aligned}$$

Following the total stress convention, equations S6 - S8, total principal stresses are given by $\sigma_1 = \sigma_H = \sigma_{H,ini} + \Delta\sigma_x$, $\sigma_2 = \sigma_v = \sigma_{v,ini} + \Delta\sigma_z$, $\sigma_3 = \sigma_h = \sigma_{h,ini} + \Delta\sigma_x$. Thus, $\Delta\sigma_m = \Delta\sigma_x$ and $\Delta\sigma_d = 0$.

In the strike-slip regime (Fig. S5B), pressure perturbations are larger than absolute changes of the mean stress if the pressure diffusion reaches a significant level at the considered location. The change of the differential stress is zero throughout the medium for all time steps, caused by the condition of plane strain with $\Delta\sigma_H = \Delta\sigma_h$. Therefore, ΔFCS is negative as long as $\left(\Delta\sigma_m - \Delta p\right) < 0$. As soon as $|\Delta p| > |\Delta\sigma_m|$, ΔFCS turns positive (Fig. 3C). The destabilisation front concept further supports this observation, yielding a front which evolves rapidly with time from the TOB to deeper intervals (Fig. 3D).

Thrust Faulting

Last, we assume a thrust faulting regime with

$$\sigma_{1,ini} = \sigma_{H,ini} = 1.25\sigma_{v,ini}$$
$$\sigma_{2,ini} = \sigma_{h,ini} = 1.05\sigma_{v,ini}$$
$$\sigma_{3,ini} = \sigma_{v,ini}$$

Total principal stresses are given by $\sigma_1 = \sigma_H = \sigma_{H,ini} + \Delta\sigma_x$, $\sigma_2 = \sigma_v = \sigma_{v,ini} + \Delta\sigma_z$, $\sigma_3 = \sigma_h = \sigma_{h,ini} + \Delta\sigma_x$, using the total stress convention, equations S6 - S8. From this it follows that $\Delta\sigma_d = \Delta\sigma_x - \Delta\sigma_z$, i.e. $\Delta\sigma_d < 0$, and $\Delta\sigma_m = \Delta\sigma_x + \Delta\sigma_z$. Generally, decreasing differential stress means a stabilisation of the medium as long as the decline is not compensated by decreasing effective normal stresses.

From this it follows for the thrust faulting setting (Fig. S5C) that the medium destabilises ($\Delta FCS > 0$) if $|0.5\Delta\sigma_d| > |\sin(\varphi)(\Delta\sigma_m - \Delta p)|$ (Fig. 3E). In the scenario of URIS discussed here, the destabilisation is time-dependent, demonstrated also by the destabilisation front (Fig. 3F).

S4. Sensitivity Study

To test the influence of parameters on ΔFCS, we performed a local, one-at-a-time (OAT) sensitivity study. While changing one parameter incrementally, the other parameters are held constant at their base level value. The great advantage of the method is that the influence of each parameter on the output can be determined directly.

For five hydraulic and elastic parameters we increased the parameter value by +10% within the interval [-50%, 50%]. To allow for stronger variations of the hydraulic diffusivity[11], the value for D was varied on a logarithmic scale between 1e-03 and 1e+03.

The influence on ΔFCS is also expressed in terms of the destabilisation front (Supplementary Fig. S6). Destabilisation fronts obtained for the base level parameter values are marked by the bold black line. Blue to red lines mark the front for parameter variations between -50% and + 50%. As was to be expected, the higher the value of ΔFCS at depth, the further the destabilization front perturbs the medium. As most of the parameters are proportional to ΔFCS (Fig. 4), the fronts evolve faster with increasing variations (Fig. S6, panels A, B, C, E, F). However, as it is inversely proportional to the basement porosity, the destabilised

domain reduces within the model time for higher Φ (Fig. S6D).

To complement the study, we tested the influence of the injection pressure p_0 under various stress regimes. Changing the positive value of p_0 (injection) to negative (discharge), the medium is stabilised in a strike-slip setting (Figs. S7A and S8), red line. Under thrusting conditions (Fig. S8, green line) stabilisation occurs only in vicinity to the injector (e.g. Fig. S8, A and E). At greater depths (Fig. S8, C - D), the medium is destabilised. However, this observation changes with time (Fig. S8, G - H), as soon as the local pore-fluid pressure increases due to diffusion (compare also to Figure S7).

Supplementary Figures

Supplementary Table

Table 1. Hydro-mechanical parameter

Parameter	Value
D	0.05 (m^2/s)
Φ	1 (%)
Φ_{Ar}	20 (%)
ρ_f	940.3 (kg/m^3)*
η	2e-04 (Pas)*
K_f	2 (GPa)*
ρ_{dr}	2740 (kg/m^3)†
G_{dr}	25 (GPa)†
λ_{dr}	20 (GPa)
α	0.3
μ_f	0.7

*[14], †[15]

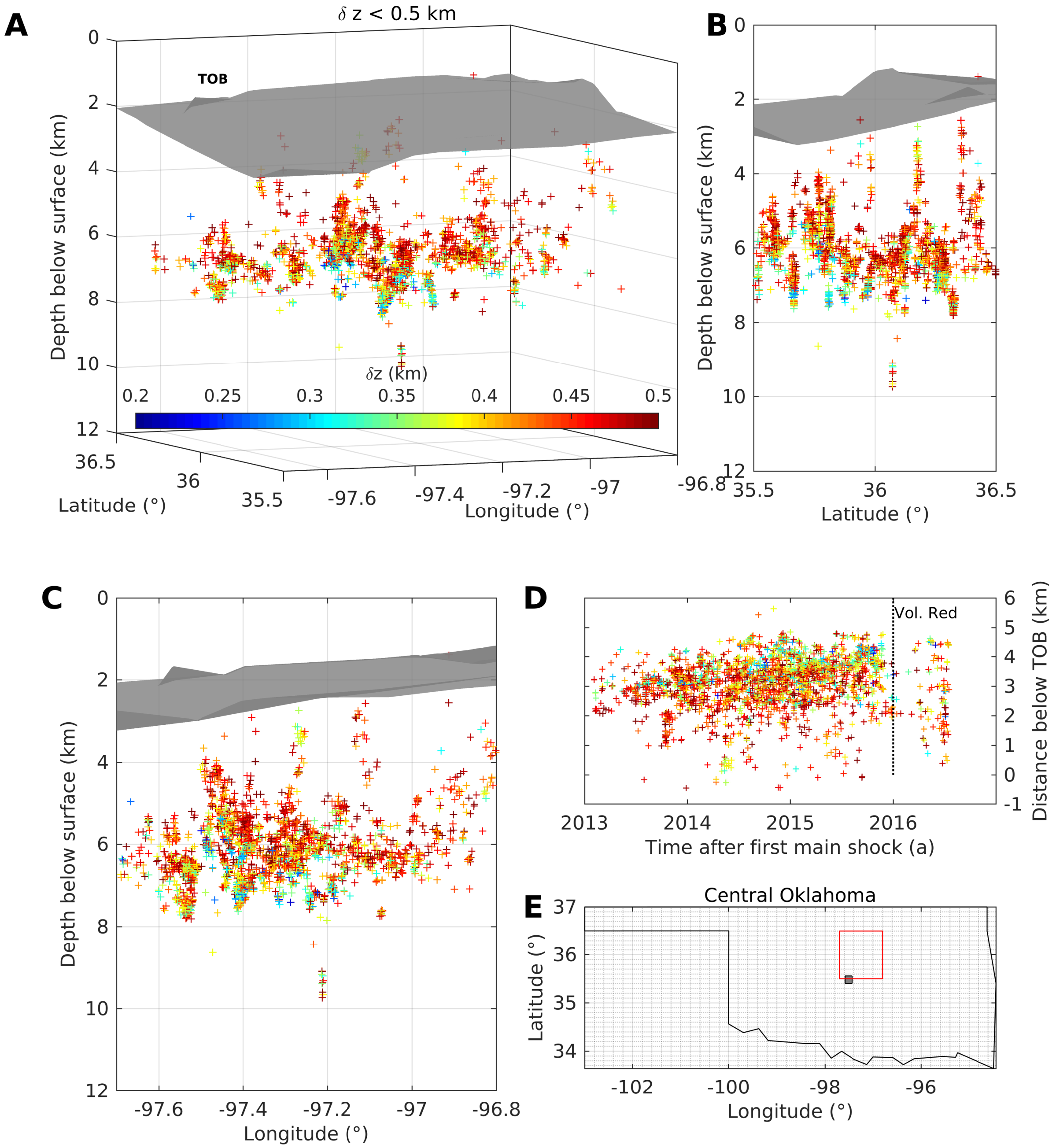

Figure S1. Seismicity in Central Oklahoma from May 2013 to November 2016. The map **(E)** demonstrates the area of interest in Central Oklahoma, bounded by latitude 35.5° to 36.5° and longitude -97.7° to -96.8°. Panels **(A)** - **(D)** show event depths and the top of the basement (TOB) relatively to the ground surface elevation. In panel **(D)**, event distances below the TOB are plotted versus their occurrence time. Year 0 denotes the time of the first event included in the catalogue on May 5, 2013. Event locations were published by Schoenball and Ellsworth[1], showing only events with depth errors $\delta z < 0.5$ km as used for the declustering. The depth of the TOB was derived from well data[12].

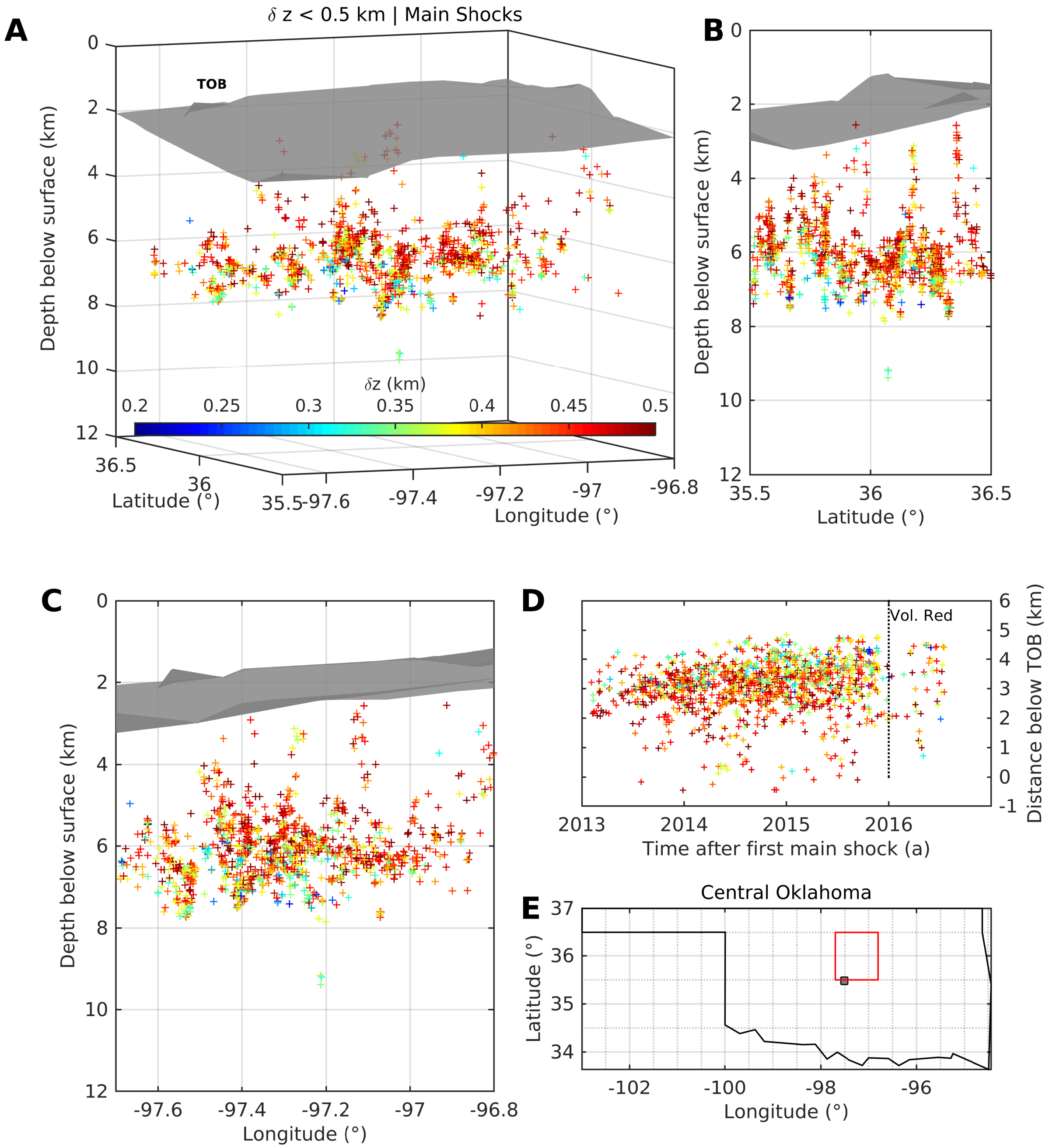

Figure S2. **Main shocks of seismicity in Central Oklahoma from May 2013 to November 2016.** Same as Figure S1 but showing only the obtained main shocks. Data by[1]

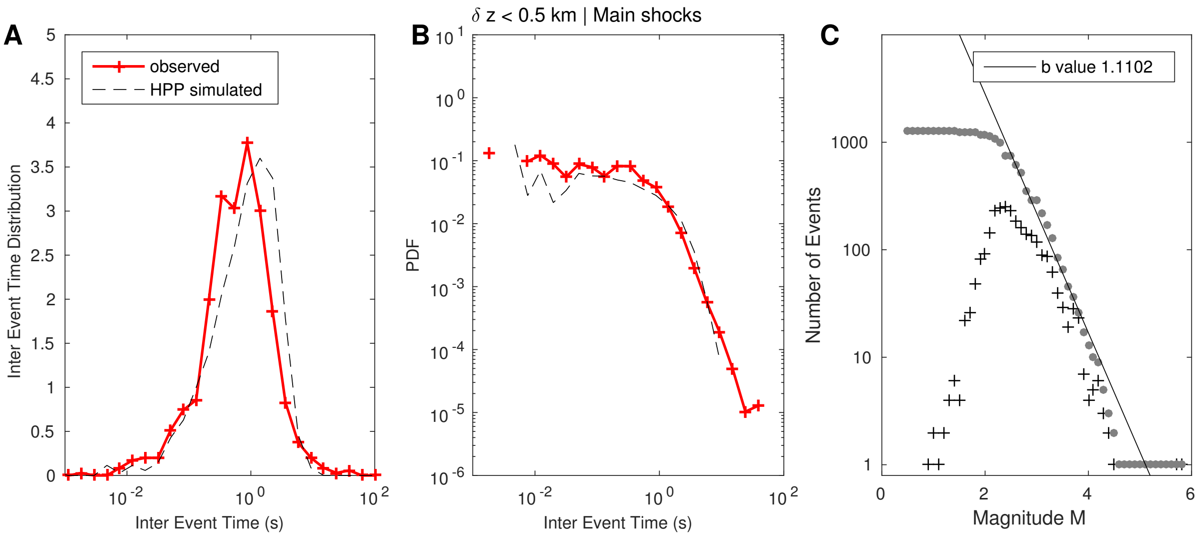

Figure S3. Inter event times and Gutenberg-Richter representation of the identified main shocks. Using a declustering algorithm, dependent events were removed from the catalogue. According to previous works, main shocks should follow a homogeneous Poisson process[2, 13]. As shown in panels **(A)** - **(B)**, the identified mains shocks are distributed as expected. Panel **(C)** is a classical Gutenberg-Richter plot.

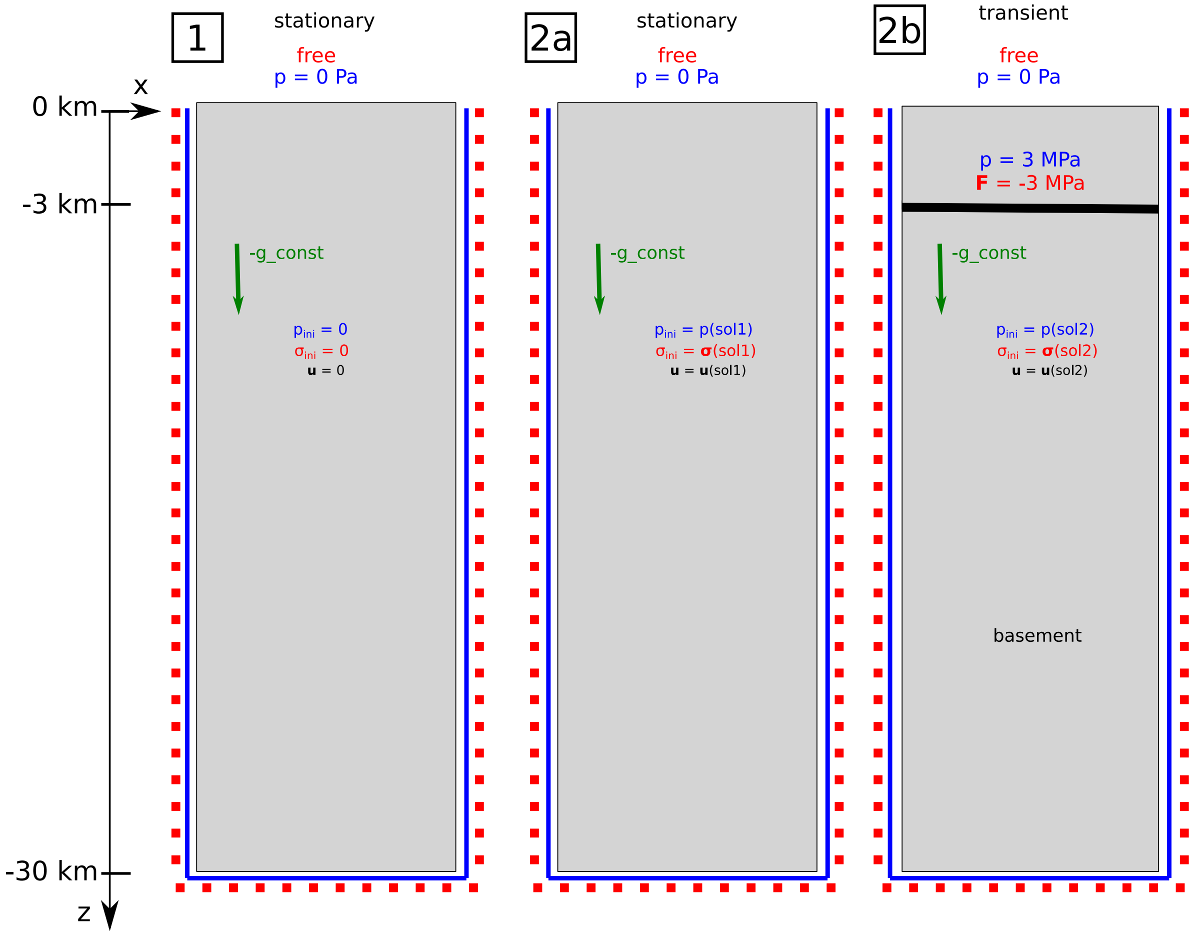

Figure S4. Geometry and boundary conditions (BC) in the numerical model. For the application of 2D plane strain, we define a block that extends in the vertical z-direction. Regarding hydrological BC, The outer boundaries are given by no flow (blue line), whereas the pressure at the top boundary is set to 0 Pa. For the elastic BC, we use so-called roller boundaries (red dotted line), i.e. movement normal to the boundaries is permitted. The top boundary is given by a free surface condition. To compute the transient solution **(2b)**, two stationary solutions **(1)** and **(2a)** are necessary which yield the initial conditions for the transient model.

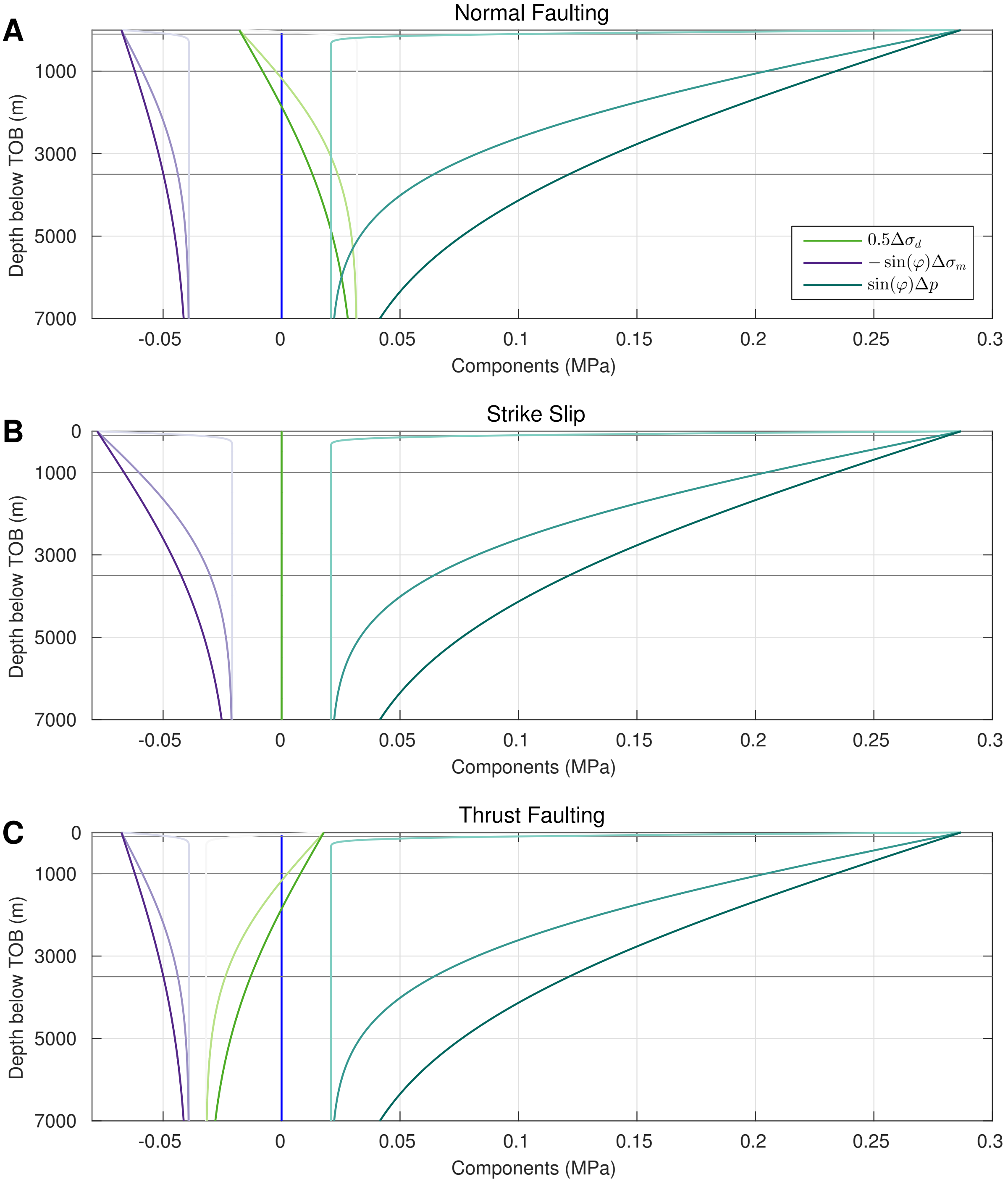

Figure S5. Components of ΔFCS for different tectonic regimes, shown for different time steps (light to dark) as profiles along the depth. While the magnitude of the components $0.5\Delta\sigma_d$ (magenta) and $-\sin(\varphi)\Delta\sigma_m$ (green) is affected by the tectonic setting (see text for discussion), $\sin(\varphi)\Delta p$ (blue) is independent of the stress regime. $0.5\Delta\sigma_d$ is zero throughout the medium in a strike-slip regime (**B**), but its value changes from negative at the TOB (stabilisation) to positive (destabilisation) at depth in a normal faulting environment (**A**). In a thrust faulting regime (**C**) it is vice versa. In contrast, the component $0.5\Delta\sigma_d$ is negative (stabilisation) in all regimes while $\sin(\varphi)\Delta p$ is positive independently of the regime. The sum of all components yields ΔFCS. Compare to Figure 4.

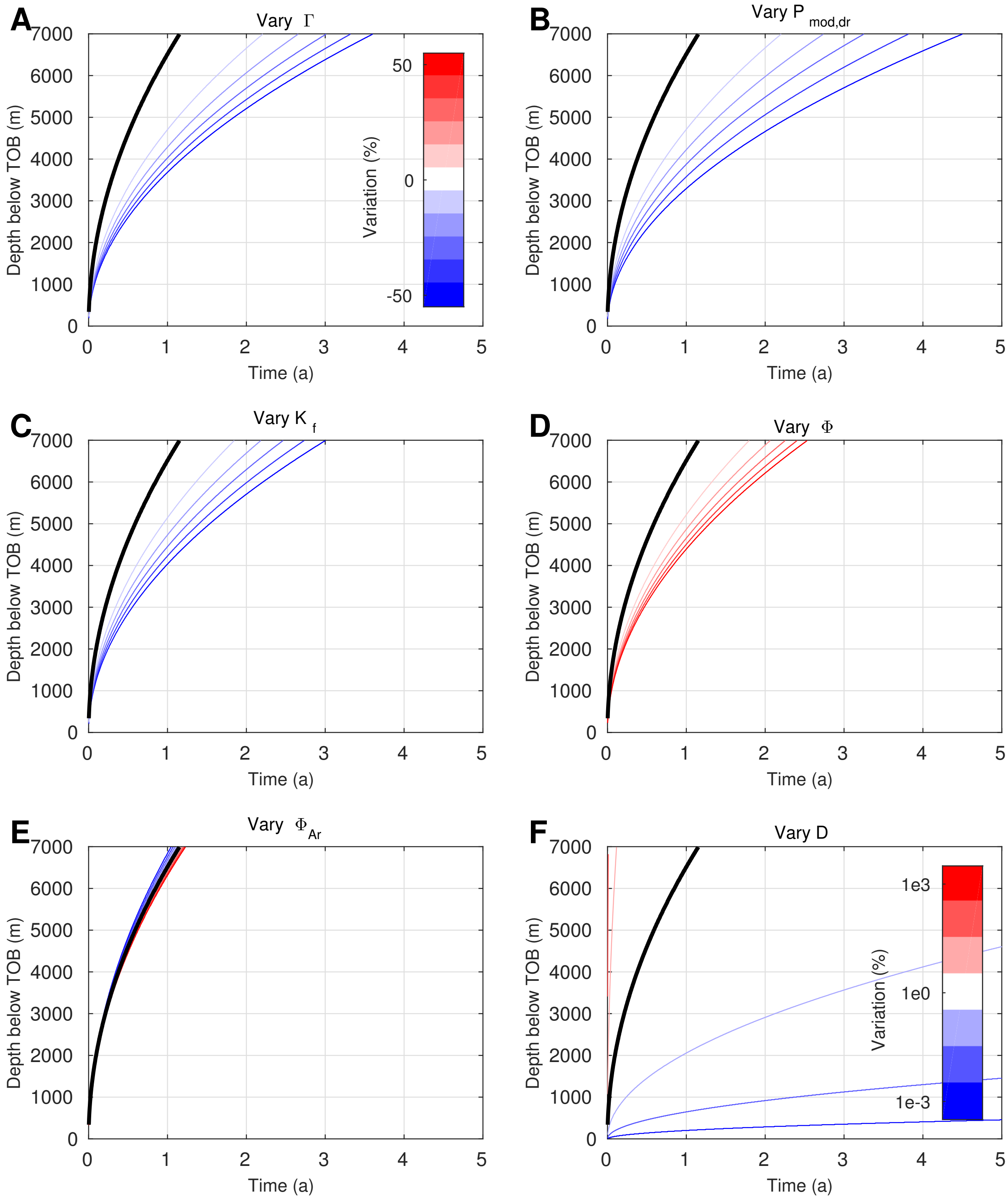

Figure S6. Parameter dependence of the destabilisation front. Blue to red lines mark smaller to higher parameter values. Fronts, obtained for the base level values as used in the modeling are depicted by the solid black line. Parameters Γ **(A)**, $P_{mod,dr}$ **(B)**, K_f **(C)** and D **(F)** are positively correlated to the evolution of the destabilisation front. The higher the parameter, the faster the front penetrates the medium. In contrast, increasing Φ **(D)** and Φ_{Ar} **(G)** lead to a slower penetration of the front.

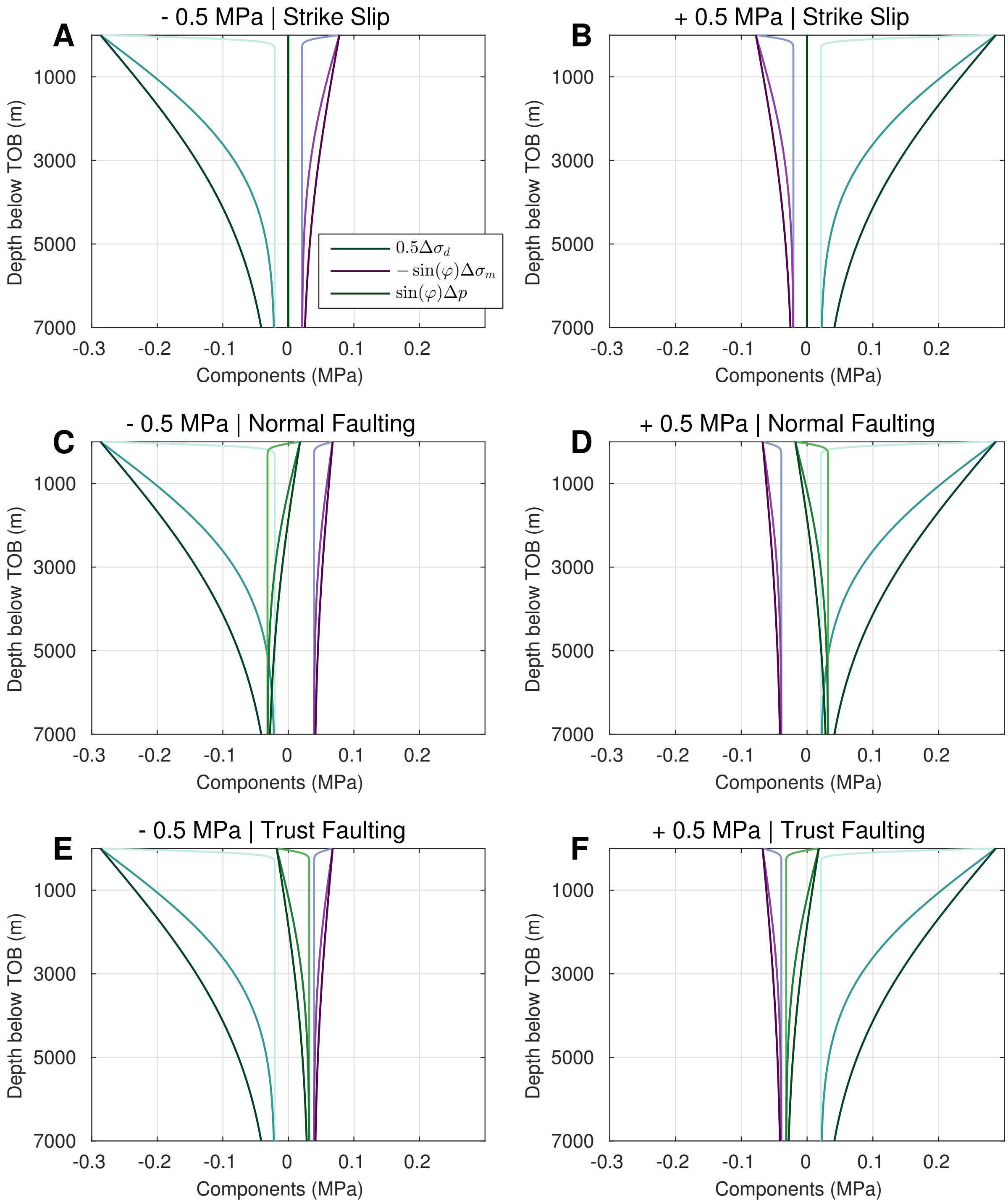

Figure S7. Components of ΔFCS for different tectonic regimes. Same as Fig. S5, but for negative (left) and positive values (right) of p_0, i.e. fluid reduction and injection, respectively. The figure points to the importance of considering poroelastic coupling in the URIS model. The sum of all three components yields an estimate of ΔFCS, shown in Figure S8.

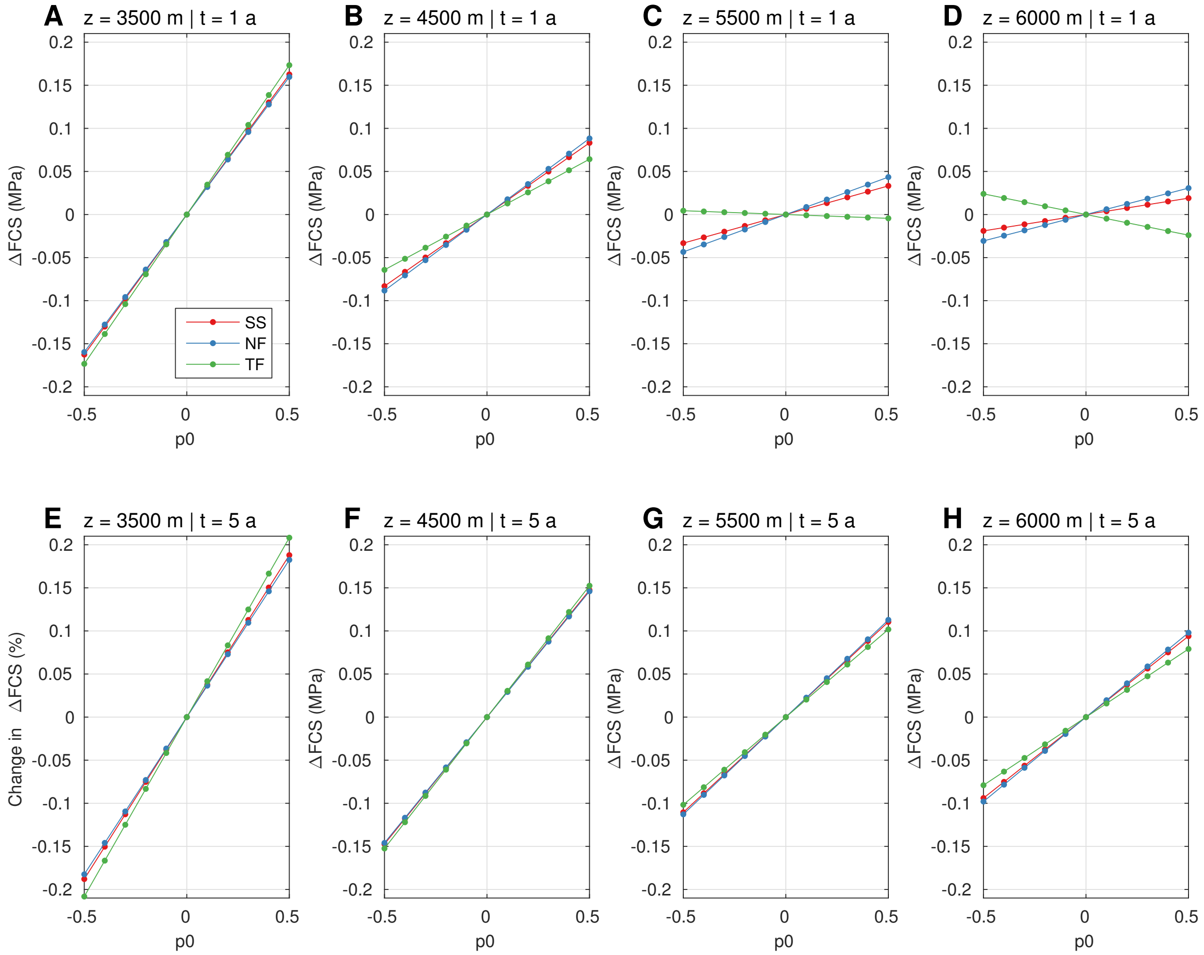

Figure S8. **ΔFCS for varying p_0 in different stress regimes.** Dependent on the time (top and bottom) and depth (from left to right), the value of ΔFCS is also controlled by the tectonic regime and the assumption of positive or negative p_0, i.e. fluid injection or reduction, respectively. In case of fluid injection in a strike-slip regime (red), the medium is destabilised most in vicinity to the injection layer (**A**). This effect weakens with distance but intensifies with time (compare **D** and **H**). In a thrust faulting environment (green), even though the medium is also destabilised close to the injection formation (**A**) but significantly stabilised at greater depth **D**. Yet, as soon as the pore pressure rises due to diffusion, destabilisation also occurs far from the injector **H**. In contrast, the situation is different under negative pressures (i.e. fluid production). Whereas locations close to as well as far away from the upper boundary are stabilised under strike-slip conditions (red line in **A** - **D**), locations at greater depth are destabilised in a thrust faulting regime (green line in **D**). Such an effect is attributable to poroelastic coupling as the main controlling factor at greater depth. As soon as pressure diffusion has reached these locations, the medium is also destabilised under thrusting conditions (**H**).

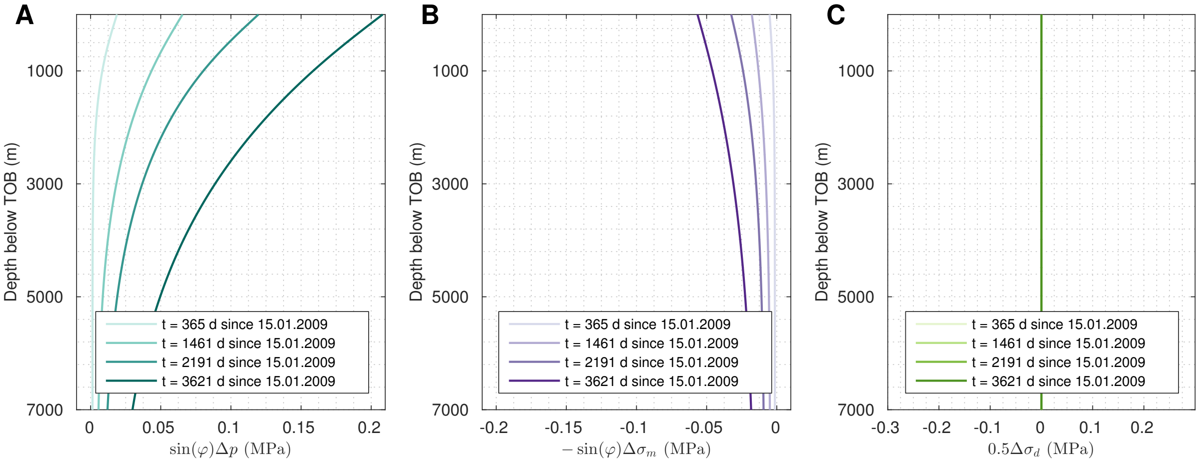

Figure S9. **Components of ΔFCS for the time-dependent boundary condition.** These are $sin\varphi\Delta p$ **(A)**, $-sin\varphi\Delta\sigma_m$ **(B)** and $0.5\Delta\sigma_d$ **(C)**. Assuming a background strike-slip regime and equal horizontal stress magnitudes $\Delta\sigma_H = \Delta\sigma_h$, the change of the failure stress is given by $\Delta FCS = sin\varphi(\Delta p - \Delta\sigma_3)$, where $\Delta\sigma_3$ is the change of the minimum principal stress and Δp is the pressure perturbation.

A

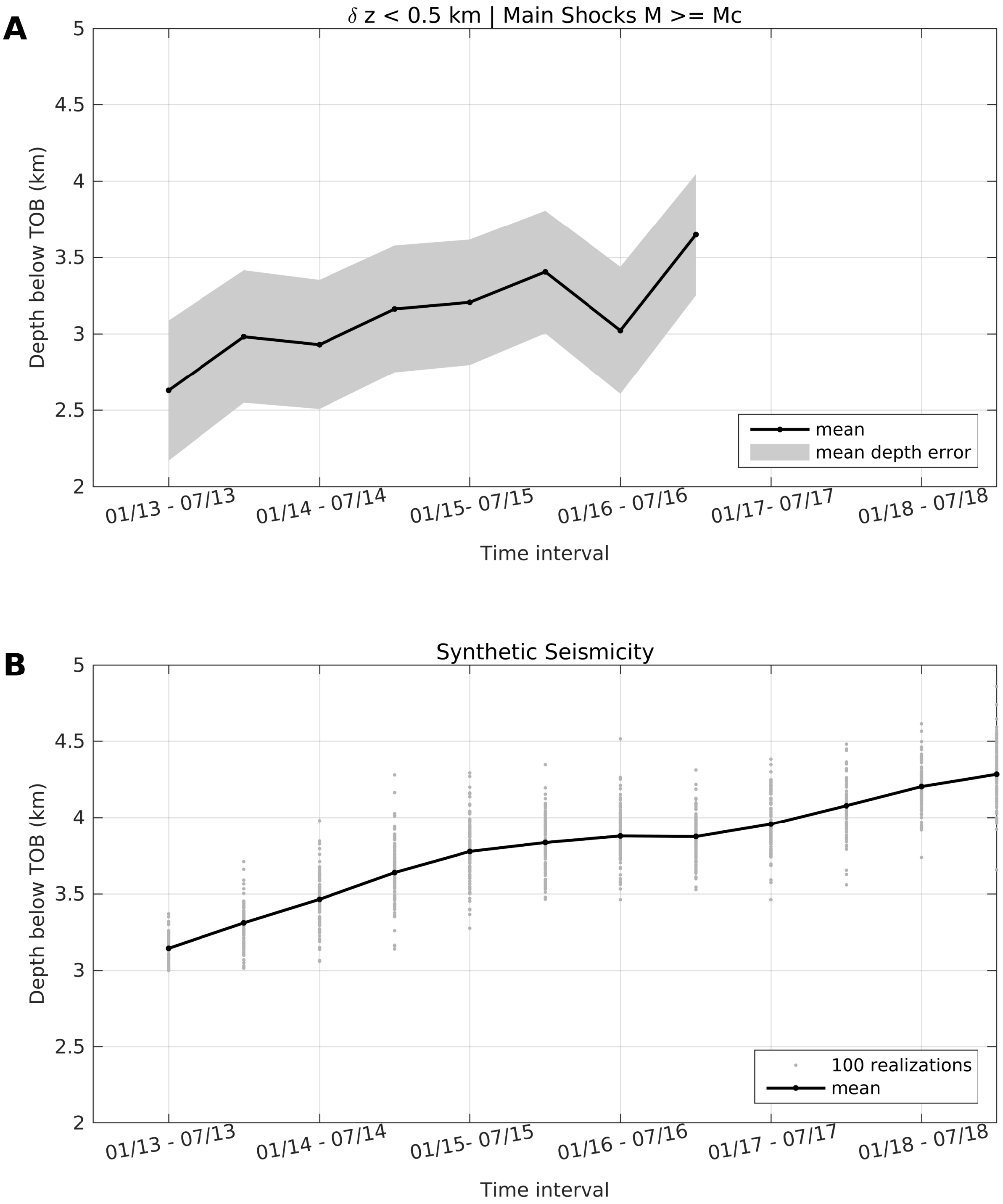

Figure S10. **Components of ΔFCS for the time-dependent boundary condition.** These are $sin\varphi\Delta p$ **(A)**, $-sin\varphi\Delta\sigma_m$ **(B)** and $0.5\Delta\sigma_d$ **(C)**. Assuming a background strike-slip regime and equal horizontal stress magnitudes $\Delta\sigma_H = \Delta\sigma_h$, the change of the failure stress is given by $\Delta FCS = sin\varphi(\Delta p - \Delta\sigma_3)$, where $\Delta\sigma_3$ is the change of the minimum principal stress and Δp is the pressure perturbation.

References

1. Schoenball, M. & Ellsworth, W. Waveform-Relocated Earthquake Catalog for Oklahoma and Southern Kansas Illuminates the Regional Fault Network. *Seism. Res. Lett.* **88** (2017). DOI 10.1785/0220170083.

2. Ogata, Y. Space-time point-process models for earthquake occurrences. *Annals Inst. Stat. Math.* **50**, 379–402 (1998). DOI 10.1023/A:1003403601725.

3. Schoenball, M. & Ellsworth, W. L. A Systematic Assessment of the Spatiotemporal Evolution of Fault Activation Through Induced Seismicity in Oklahoma and Southern Kansas. *J. Geophys. Res. Solid Earth* **122**, 10189–10206 (2017). DOI 10.1002/2017JB014850.

4. Urhammer, R. A. Characteristics of northern and central california seismicity. *Earthq. Notes* **57**, 21 (1986).

5. Gutenberg, B. & Richter, C. F. Frequency of earthquakes in California. *Bull. Seism. Soc. Am.* **34**, 185–188 (1944).

6. Shapiro, S. *Fluid-Induced Seismicity* (Cambridge Univerisity Press, Cambridge, U.K., 2015).

7. Detournay, E. & Cheng, A. *Fundamentals of Poroelasticity*, vol. 2 (Pergamon Press, 1993).

8. Snow, D. Geodynamics of seismic reservoirs. In *Symposium on Percolation Through Fissured Rock*, T2 – J: 1 – 19 (Deutsche Gesellschaft für Erd- und Grundbau, Germany, 1972).

9. Shapiro, S. A. & Dinske, C. Scaling of seismicity induced by nonlinear fluid-rock interaction. *J. Geophys. Res. Solid Earth* **114**, 14pp (2009). DOI 10.1111/j.1365-2478.2008.00770.x.

10. Hummel, N. & Shapiro, S. A. Microseismic estimates of hydraulic diffusivity in case of non-linear fluid-rock interaction. *Geophys. J. Int.* **188**, 1441–1453 (2012). DOI 10.1111/j.1365-246X.2011.05346.x.

11. Langenbruch, C. & Shapiro, S. Gutenberg-Richter relation originates from Coulomb stress fluctuations caused by elastic rock heterogeneity. *J. Geophys. Res. Solid Earth* **119** (2014). DOI 10.1002/2013JB010282.

12. Campbell, J. A. & Weber, J. L. Wells drilled to Basement in Oklahoma. *Okla. Geol. Surv. Special Publ.* **1** (2006).

13. Langenbruch, C., Dinske, C. & Shapiro, S. A. Inter event times of fluid induced earthquakes suggest their Poisson nature. *Geophys. Res. Lett.* **38** (2011). DOI 10.1029/2011GL049474. L21302.

14. Norbeck, J. H. & Horne, R. N. Evidence for a transient hydromechanical and frictional faulting response during the 2011 Mw 5.6 Prague, Oklahoma earthquake sequence. *J. Geophys. Res. Solid Earth* **121**, 8688–8705 (2016). DOI 10.1002/2016JB013148.

15. Chang, K. W. & Segall, P. Injection-induced seismicity on basement faults including poroelastic stressing. *J. Geophys. Res. Solid Earth* **121**, 2708 – 2726 (2016). DOI 10.1002/2015JB012561.
